# The structure of kaliophilite KAlSiO_4_, a long-lasting crystallographic problem

**DOI:** 10.1107/S2052252520012270

**Published:** 2020-09-29

**Authors:** Enrico Mugnaioli, Elena Bonaccorsi, Arianna E. Lanza, Erik Elkaim, Virginia Diez-Gómez, Isabel Sobrados, Mauro Gemmi, Miguel Gregorkiewitz

**Affiliations:** a Center for Nanotechnology Innovation@NEST, Istituto Italiano di Tecnologia, Piazza S. Silvestro 12, Pisa, 56127, Italy; b Dipartimento di Scienze della Terra, Università di Pisa, Via Santa Maria 53, Pisa, 56126, Italy; c Synchrotron Soleil, L’Orme des Merisiers, Saint-Aubin, Gif-sur-Yvette, 91192, France; d Instituto de Ciencia de Materiales de Madrid, Consejo Superior de Investigaciones Científicas (ICMM-CSIC), Sor Juana Inés de la Cruz 3, Madrid, 28049, Spain; e Dipartimento di Scienze Fisiche, della Terra e dell’Ambiente, Università di Siena, Via Laterina 8, Siena, 53100, Italy

**Keywords:** framework silicates, electron diffraction, framework topologies, microstructures, kaliophilite, structure refinement

## Abstract

Kaliophilite is a feldspathoid mineral found in two Italian magmatic provinces and represents one of the 12 known phases with composition close to KAlSiO_4_. Despite its apparently simple formula, the structure of this mineral revealed extremely complex and resisted structure solution for more than a century. Samples from the Vesuvius–Monte Somma and Alban Hills volcanic areas were analyzed through a multi-technique approach, and finally the crystal structure of kaliophilite was solved using 3D electron diffraction and refined against X-ray diffraction data of a twinned crystal. Results were also ascertained by the Rietveld method using synchrotron powder intensities. It was found that kaliophilite crystallizes in space group *P*3 with unit-cell parameters *a* = 27.0597 (16), *c* = 8.5587 (6) Å, *V* = 5427.3 (7) Å^3^ and *Z* = 54. The kaliophilite framework is a variant of the tridymite topology, with alternating SiO_4_ and AlO_4_ tetrahedra forming sheets of six-membered rings (6^3^ nets), which are connected along [001] by sharing the apical oxygen atoms. Considering the up (U) and down (D) orientations of the linking vertex, kaliophilite is the first framework that contains three different ring topologies: nine (1-3-5) (UDUDUD) rings, six (1-2-3) (UUUDDD) rings and twelve (1-2-4) (UUDUDD) rings. This results in a relatively open (19.9 tetrahedra nm^−3^) channel system with multiple connections between the double six-ring cavities. Such a framework requires a surprisingly large unit cell, 27 times larger than the cell of kalsilite, the simplest phase with the same composition. The occurrence of some Na for K substitution (3–10%) may be related to the characteristic structural features of kaliophilite. Micro-twinning, pseudo-symmetries and anisotropic *hkl*-dependent peak broadening were also detected, and they may account for the elusive character of the kaliophilite crystal structure.

## Abstract 2

The elusive structure of the mineral kaliophilite has been determined by 3D electron diffraction and refined using single-crystal X-ray data. Despite its simple formula, ideally KAlSiO_4_, the structure of this mineral remained a mystery for over a century as a result of pseudo-symmetry and twinning, which reduce the coherent crystalline domain size to a few hundreds of nanometres.

## 1. Introduction   

Kaliophilite is a rare mineral of the feldspathoid (framework silicate) group, up to now only known from Italian potassic to ultrapotassic magmatic provinces, namely the Monte Somma, Somma–Vesuvius complex, Naples province, Campania, Italy (type locality; Mierisch, 1887 ▸) and several locations of the Roman Magmatic Province, Lazio, Italy (Colli Albani, Barbieri *et al.*, 1970 ▸; Di Battistini *et al.*, 2001 ▸). It was the first to be identified among 12 phases with compositions near to KAlSiO_4_. At first, it was thought to be the potassium end-member in a simple isomorphous series with nepheline at the Na-rich side, both being hexagonal. However, with the advent of X-ray diffraction, things proved to be considerably more complicated: other phases in the (K, Na)AlSiO_4_ series were discovered (Bannister & Hey, 1942 ▸; Sahama & Smith, 1957 ▸; Smith & Tuttle, 1957 ▸; Cook *et al.*, 1977 ▸; Minor *et al.*, 1978 ▸; Franco & De Gennaro, 1988 ▸; Cellai *et al.*, 1997 ▸; Khomyakov *et al.*, 2002 ▸), six crystal structures of increasing complexity were resolved and it eventually became clear that kalsilite (and not kaliophilite) is the actual potassium end-member of the series. Both kalsilite and nepheline are in fact stuffed derivatives of high-tridymite topology ([Si_2_O_4_]; Buerger, 1954 ▸). Their framework contains sheets of six-membered rings (6^3^ nets; Smith, 1977 ▸) of tetrahedra, connected along [001] by sharing the apical oxygen atoms in a ring-over-ring stacking. In kalsilite and nepheline all rings of tetrahedra have vertices pointing alternatively up (U) and down (D), usually indicated as UDUDUD or, more briefly, (1-3-5). Other kinds of rings, such as UUUDDD or (1-2-3) and UUDUDD or (1-2-4) (see Fig. S1.1 in the Supporting information), are known to occur, giving rise to several variants of the tridymite topology (Dollase, 1969 ▸). All phases with these topologies and composition close to KAlSiO_4_ (including nepheline) are listed in Table 1 ▸.

Beyond geology and mineralogy, interest in structures based on the tridymite topology and its variants comes from materials science. A rich variety of framework topologies and conformations, combined with a broad choice of compositions, makes them ideal for molecular engineering. They have been used to study ferroics and critical phenomena (Hildmann, 1980 ▸; Hammonds *et al.*, 1996 ▸; Nénert *et al.*, 2013 ▸), and to explore the mechanisms of ion exchange and conduction in solid electrolytes (Gregorkiewitz, 1986 ▸; Jiménez & Gregorkiewitz, 1999 ▸; Jones *et al.*, 2001 ▸). In particular, K[AlSiO_4_] compounds play an important role in refractory materials (Cook *et al.*, 1977 ▸) and in the formation of agglomerates in fluidized bed reactors (Wang *et al.*, 2018 ▸). More recently, their catalytic action in the abatement of diesel soot (Becerro *et al.*, 2009 ▸; Li *et al.*, 2014 ▸) and in the production of biodiesel was recognized (He *et al.*, 2019 ▸).

Kaliophilite itself has never been obtained in the laboratory. Based on cation-exchange experiments, Gregorkiewitz (1986 ▸) inferred that natural hexagonal kaliophilite has a different topology from any other phase of the KAlSiO_4_ system, and in particular from stuffed tridymite phases such as nepheline and kalsilite. Attracted by the peculiarities of kaliophilite diffraction features, many researchers have attempted to decipher the enigma of its structure for more than 100 years without success. Only the recent establishment of the three-dimensional electron-diffraction (3D ED) method (Mugnaioli & Gemmi, 2018 ▸; Gemmi *et al.*, 2019 ▸) allowed the structure determination of kaliophilite, its successive X-ray diffraction refinement and a proper understanding of how pseudo-symmetry and twinning operate at the nanoscale. Remarkably, kaliophilite presents a new topology based on three different ring types, different from those hitherto known for KAlSiO_4_ compounds and for all other materials based on tridymite topology and its variants.

### 1.1. Previous studies on kaliophilite   

The first occurrence of kaliophilite inside Vesuvius blocks was described by Covelli (1839) ▸, who correctly listed its main physical and chemical properties (see Table S1.1 in the Supporting information) and called it ‘beudantina’. Later, several authors discredited the mineral, considering it a mere variety of nepheline. It took 50 more years before Mierisch (1887 ▸) described a colorless prismatic mineral in the ejected blocks of the Vesuvius–Monte Somma volcanic complex. He found that this mineral was optically uniaxial negative, with a density of 2.602 g cm^−3^ and probably hexagonal symmetry. He also hypothesized that the mineral was isomorphous with nepheline and suggested the name kaliophilite for its elevated potassium content.

The unit cell of kaliophilite was later investigated by Gossner & Mussgnug (1930 ▸), who found two possible hexagonal unit cells, with *a* = 27 Å and *a* = 15.6 Å, respectively, and *c* = 8.6 Å. They observed extinctions for 00*l* with odd *l* but were reportedly unable to test for the *c* glide (extinction condition *h*0*l*: *l* = 2*n* + 1). A weak intensity for the 005 reflection was reported anyway, hindering a definitive symmetry determination. Natural kaliophilite was also studied by Bannister & Hey (1931 ▸), who redetermined density and optical indices and pointed out that the unit cell of kaliophilite is indeed surprisingly large with a volume 27 times that of tridymite. Lukesh & Buerger (1942 ▸) suggested the diffraction symbol 6/*mmmP*6_3_-- (possible space group *P*6_3_22) and inferred the absence of the inversion center based on piezo and pyroelectric effects. Eventually, Claringbull & Bannister (1948 ▸) proposed that the crystal structure of kaliophilite was a multiple of the kalsilite structure. Invoking twinning, Buerger (1954 ▸) questioned space group *P*6_3_22, but since then many textbooks and databases have accepted the idea without further comment.

Hexagonal kaliophilite has never been synthesized. A pseudo-orthorhombic phase was obtained by different authors anyway (Lemberg, 1876 ▸; Duboin, 1892 ▸; Bowen, 1917 ▸; Rigby & Richardson, 1947 ▸; Kunze, 1954 ▸). A systematic study of different possible synthetic polymorphs was carried out by Smith & Tuttle (1957 ▸). Besides kalsilite, they obtained two other hexagonal phases [*H*4 and ‘synthetic kaliophilite’, the latter also referred to as *H*2 by Merlino (1984 ▸)] and two apparently orthorhombic phases, *O*1 and *O*2, where *O*1 is identical with the pseudo-orthorhombic material mentioned above (Table 1 ▸). It appeared that the ‘orthorhombic’ phases were stable at higher temperatures. Cook *et al.* (1977 ▸) also observed another phase (*O*1-hT) above 1450°C.

The *O*1 phase was eventually shown to be monoclinic (Gregorkiewitz, 1980 ▸), with a new topology based on two different kinds of rings, (1-2-3) and (1-2-4). Its crystal structure was finally refined in space group *P*2_1_ using a combination of X-ray powder diffraction and ^29^Si magic angle spinning nuclear magnetic resonance (MAS–NMR) data (Gregorkiewitz *et al.*, 2008 ▸), and from twinned single-crystal X-ray diffraction (Kremenović *et al.*, 2013 ▸). A natural occurrence of *O*1 was recently reported in the Hatrurim Complex, Negev Desert, Israel (Krüger *et al.*, 2016 ▸).

## 2. Experimental   

### 2.1. Samples and chemical composition   

Various kaliophilite samples were used. Three samples were from different excavation sites on Colle Cimino, Alban Hills near Rome (cc1, cc2 and cc3), and two were from the volcanic area of Vesuvius–Monte Somma, namely from the Pollena and Monte Somma locations, ms1 and ms2, respectively. The elemental composition of the cc1 and cc2 samples was determined from energy-dispersive X-ray spectra obtained using a JEOL JXA840 scanning electron microscope at CENIM-CSIC, working at 15 kV and with take-off angle 40°, and an ISIS Oxford spectrometer mounted on a JEOL 2010 transmission electron microscope, working at 200 kV, respectively. Quantitative chemical analyses of the cc3, ms1 and ms2 samples were carried out using a Superprobe JEOL JXA 8200 electron microprobe (wavelength-dispersive spectroscopy mode, 15 kV, 10 nA, 1 µm beam diameter) at the Eugen F. Stumpfl laboratory, Leoben University, Austria. The standards were albite (Na), sanidine (K, Si, Al), wollastonite (Ca) and olivine (Fe).

### 2.2. MAS–NMR spectroscopy and density functional theory calculations   

About 200 mg of carefully selected and gently crushed kaliophilite crystals from cc1 and cc2 were purified in a Kranz magnetic separator to eliminate traces of Fe-containing impurities and compacted in a 4 mm ZrO_2_ rotor.

High-resolution solid-state ^29^Si and ^27^Al MAS–NMR spectra (see section S2 in the Supporting information) were recorded at 79.49 and 104.23 MHz (9.4 T magnetic field), respectively, on a Bruker Avance 400 spectrometer equipped with a Fourier transform unit while spinning (10 kHz) the sample at the magic angle (54°44′). The pulse lengths were 6 and 2 µs and the recycle delays were 10 and 5 s for the ^29^Si and ^27^Al nuclei, respectively, to maximize the intensity of the experimental signal. The number of accumulations was 800 and 200 for the Si and Al signals, respectively. Chemical shifts δ (in p.p.m.) are given relative to tetra­methyl­silane (for ^29^Si) and 1 *M* of AlCl_3_ aqueous solution (for ^27^Al) as external standards. The spectra were fitted with pseudo-Voigtian peak shapes using the program *Winfit* (Bruker; Krumm, 1996 ▸), based on a non-linear least-squares iterative method.

The calculation of chemical shifts as a function of structure was carried out using the CASTEP 2017R2 code (Clark *et al.*, 2005 ▸), which makes use of density functional theory (DFT). The gauge-including projector augmented-wave (GIPAW) algorithm (Pickard & Mauri, 2001 ▸) and the generalized gradient approximation (GGA) Perdew–Burke–Ernzerhof (PBE) functional (Perdew *et al.*, 1996 ▸) were used, and the core–valence interactions were described with ultrasoft pseudo-potentials (Yates *et al.*, 2007 ▸). The crystal structure was reproduced by using periodic boundary conditions. Numerical integrals were performed over the Brillouin zone, using a Monkhorst–Pack grid with a *k*-point spacing of 0.07 Å^−1^. Wavefunctions were expanded in plane waves with kinetic energy smaller than the cut-off energy of 600 eV. To correlate experimental chemical shifts δ_iso_ to the calculated chemical shieldings σ_iso_, previous DFT calculations in similar compounds were used, obtaining the equation δ_iso_ = −0.6941σ_iso_ + 199.26.

### 2.3. High-resolution transmission electron microscopy observations   

Selected grains of kaliophilite from ms1 and cc3 samples were ground, suspended in iso­propyl alcohol and deposited over 400 mesh copper grids coated with carbon film. Selected-area electron diffraction (SAED) and high-resolution images were obtained using a Philips 400T transmission electron microscope operating at 120 kV. Although the crystals of kaliophilite were highly sensitive to electron-beam damage and became amorphous after exposure to the electron beam for half a minute, it was possible to obtain high-resolution images and SAEDs of kaliophilite crystals before their degradation.

### 2.4. Three-dimensional electron diffraction and *ab* *initio* model determination   

Three-dimensional ED experiments (Kolb *et al.*, 2007 ▸; Gemmi *et al.*, 2019 ▸) were performed for samples cc1 and cc2 at the Center for Nanotechnology Innovation@NEST (Istituto Italiano di Tecnologia) in Pisa, Italy, using a Zeiss Libra 120 transmission electron microscope operating at 120 kV and equipped with an LaB_6_ source. Three-dimensional ED data were collected in scanning transmission electron-microscopy (STEM) mode without using a selected-area aperture, after defocusing the beam in order to have a pseudo-parallel illumination on the sample. A beam size of ∼150 nm in diameter was obtained by inserting a 5 µm C2 condenser aperture (Gemmi & Lanza, 2019 ▸). An extremely mild illumination was adopted in order to avoid any alteration or amorphization of the sample.

Three-dimensional ED was performed with a precessing beam (Vincent & Midgley, 1994 ▸; Mugnaioli *et al.*, 2009 ▸) obtained using a Nanomegas Digistar P1000 device. The precession semi-angle was kept at 1°. In total, 116 diffraction patterns were recorded in a tilt range of 115°. The camera length was kept at 230 mm with an actual resolution of ∼1.0 Å. Data were recorded by an ASI Timepix single-electron detector able to deliver a pattern that is virtually background free. More detailed information is reported in Table S5.1.

The intensities were integrated using *ADT3D* software (Kolb *et al.*, 2011 ▸) and in-home developed *MATLAB* routines. *Ab initio* structure determination was performed by the direct methods implemented in *SIR2014* (Burla *et al.*, 2015 ▸). A preliminary refinement was performed in a standard kinematical approach using *SHELXL* (Sheldrick, 2015 ▸) with SADI restraints on Si–O and Al–O distances.

### 2.5. Laboratory single-crystal X-ray diffraction   

A single crystal of kaliophilite was selected from the ms1 sample. Intensities were collected by means of a Bruker Smart Breeze diffractometer equipped with an air-cooled CCD detector, using graphite-monochromated Mo *K*α radiation. Seven datasets of ∼300 frames were collected in 0.5° slices with an exposure time of 45 s. The detector-to-crystal working distance was set to 50 mm. Data were integrated and corrected for Lorentz and polarization factors, background effects, and absorption using *Apex* 3 (Bruker), resulting in 127 892 measured and 37 707 unique reflections in space group *P*3, with *a* = 27.0597 (16) and *c* = 8.5587 (6) Å. These intensities were used for least-squares refinements (with the program *SHELXL*; Sheldrick, 2015 ▸). Crystal information, as well as data-collection and refinement parameters, are reported in Table S5.2.

### 2.6. Synchrotron single-crystal X-ray diffraction   

For several selected microcrystals from sample cc1 (*V* ≃ 10^−4^ mm^3^, diffraction power *S* = ρ_e_^2^ *V*_c_λ^3^ ≃ 2 × 10^16^ e^2^ [where ρ_e_ = electron density (e Å^−1^), *V*_c_ = crystal volume (Å^3^), λ = wavelength (Å), e = number of electrons], high-resolution synchrotron ‘single’-crystal X-ray diffraction scans were performed on the WDIF4C four-circle goniometer at beamline W22 at DCI LURE, Orsay, France (Bessière *et al.*, 1987 ▸; Lauriat, 1986 ▸). This instrument has an angle reproducibility of less than 0.001° and includes *Q*-scan facilities with resolution δ*Q* ≃ 3 × 10^−4^ Å^−1^ (*Q* = 2sinθ/λ), the wavelength was set to λ = 0.6888 Å (Zr *K* absorption edge) using a Si(111) double-crystal monochromator (with the second crystal bent for sagittal focusing) and intensities were maximized using vacuum waveguides for both the incident and the diffracted beam. The beam size was defined by horizontal and vertical slits, the flux at the sample was ∼1 × 10^10^ photons s^−1^, and the detector was a NaI scintillation counter with saturation of ∼100 kcps (× 10^3^ counts per second).

Evaluation of the data was made using homemade programs for the extraction of peak parameters [maximum and integrated intensity, full width at half-maximum (FWHM), linear background] and graphic presentation.

### 2.7. Synchrotron powder X-ray diffraction   

A synchrotron powder X-ray diffraction pattern was obtained from sample cc1 at SLS (Villigen, Switzerland) at the MS-X04SA Materials Science beamline (Willmott *et al.*, 2013 ▸), operating in Debye–Scherrer geometry, and equipped with a MYTHEN II 120° curved position-sensitive detector (Bergamaschi *et al.*, 2010 ▸) at a distance of 784.45 mm and with an angular resolution of 0.0037°(2θ). The sample was packed in a glass capillary of 0.3 mm in diameter and kept spinning during exposure to a parallel monochromatic beam (λ = 1.18091 Å) of 0.4 × 4 mm. Diffraction data of excellent quality were obtained with a step size of 0.0018° in the range 2.5–121.0968° in 2θ, corresponding to a resolution range of *d* = 13.5–0.690 Å.

This pattern was introduced in Rietveld, Le Bail and single-peak least-squares refinements using the *FullProf* program suite (Rodríguez-Carvajal, 2001 ▸) combined with the *WinPLOTR* graphical tool (Roisnel & Rodríguez-Carvajal, 2001 ▸). The background was simulated by linear interpolation between 51 fixed points, a correction of the pattern origin and sample displacement was allowed for, and peak profiles were calculated using the Thompson–Cox–Hastings (TCH) pseudo-Voigt function (Thompson *et al.*, 1987 ▸), providing for both instrument- and sample-dependent parameters. The instrument-dependent profile parameters used [the Gaussian variances *U*, *V* and *W* in the work of Caglioti *et al.* (1958 ▸)] were found from independent Le Bail refinements of a cubic standard material (1.4 µm Na_2_Ca_3_Al_2_F_14_, ‘NAC’; Courbion & Ferey, 1988 ▸).

## 3. Results   

### 3.1. Chemical and MAS–NMR characterization   

The elemental compositions of the Colle Cimino and Vesuvius–Monte Somma samples are given in Table 2 ▸. The average alkali content is slightly different for the two provenances, with a higher Na content in the Vesuvius–Monte Somma samples (∼2 wt% of Na_2_O) with respect to the Colle Cimino samples (less than 1 wt% of Na_2_O). The mean for all samples is K_0.93_Na_0.06_Fe_0.01_Al_0.99_Si_01.01_O_4_, essentially in agreement with the work of Cellai *et al.* (1992 ▸). Earlier data often included significant amounts of calcium, but they were based on wet chemical analyses of bulk samples which might contain impurities. All published chemical analyses of kaliophilite are reported in Table S1.1.

MAS–NMR spectra show a single line for both ^29^Si and ^27^Al, with chemical shifts δ corresponding to Si(OAl)_4_ and Al(OSi)_4_, *i.e.* there is a perfect alternation of Al and Si as expected for an Al:Si = 1:1 tetrahedral framework (Loewenstein, 1954 ▸). Lines for Si(Al) are broader than expected for a single peak, thus suggesting superposition of several signals. The simulated ^29^Si peak confirms this hypothesis (see Fig. S2.1). It is composed of two relatively sharp (FWHM ≃ 5) structural peaks at the center (−86.5 and −89.5 p.p.m.), corresponding to the 18 different crystallographic environments of the final refined structural model (Table S5.3), and an important contribution of four broad peaks at the base, suggesting the presence of some impurities (−74 to −80 p.p.m.) as well as distortion (−90  p.p.m.) and Si for Al substitutions (−96 and −105 p.p.m.) in kaliophilite itself, *e.g.* at domain boundaries. For further details see section S2 in the Supporting information.

### 3.2. Synchrotron single-crystal X-ray diffraction and high-resolution transmission electron microscopy   

Kaliophilite typically consists of tiny needles welded to­gether in bunches that may reach a thickness up to 1 mm and a few millimetres in length. Single needles are rare, but ∼12 apparently pure and optically homogeneous microcrystals, ∼40 µm across, could be isolated under a polarized light microscope and further screened with laboratory single-crystal X-ray diffraction (see section S3 in the Supporting information). Many of them revealed to be composed of slightly (∼1°) misoriented individuals, resulting in split reflections.

**Figure 1:**
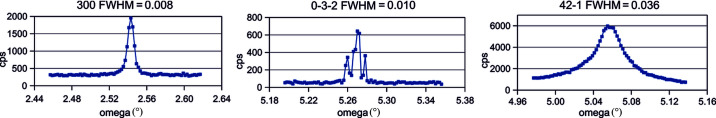
Representative high-resolution scans for three types of reflections. 300 and 0-3-2 comply with parity rule *h* − *k* = 3*n*, while 42-1 disobeys the rule. Moreover, the 0-3-2 reflection shows micro-splitting because of the misalignment of tiny kaliophilite domains. Note that the abscissa has the same scale in all cases and λ = 0.6888 Å.

In order to test symmetry and twinning, one crystal that apparently showed no splitting was used to collect high-resolution synchrotron X-ray diffraction scans for lattice equivalent reflections (see section S4 in the Supporting information). This revealed that reflections with indices *h − k* = 3*n* are systematically sharp (0.008 < FWHM < 0.012°) with approximately Gaussian shape, while the remaining reflections (*h − k* ≠ 3*n*) show FWHM ≃ 0.035° with a more Lorentzian shape (Fig. 1 ▸). The parity *h − k* = 3*n* corresponds to a subcell with *a*′ = *a*/3^1/2^ ≃ 15.6 Å. The difference in FWHM suggests that the proper 27 Å cell forms smaller coherent domains within the same crystal.

Furthermore, looking closer at the sharp reflections we detected micro-splitting at an unexpectedly fine scale, indicating that our crystal was made up of at least four domains, each with FWHM ≃ 0.004° and mutual misalignment within ∼0.02°. It therefore appears that even tiny single crystals of kaliophilite tend to a fibrous growth of multiple misaligned individuals, possibly associated with stacking faults or twin boundaries.

**Figure 2:**
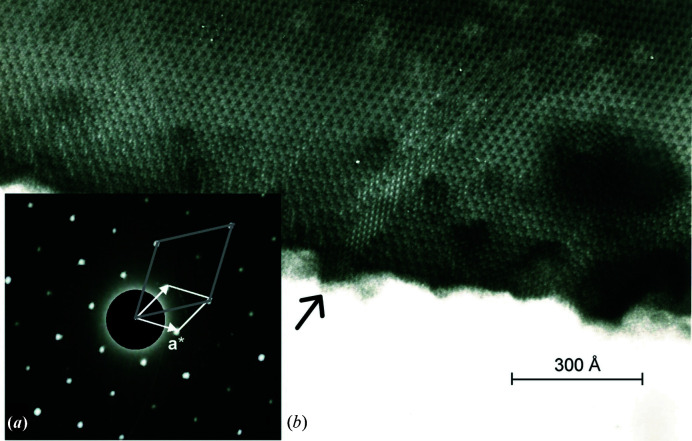
(*a*) SAED patterns of kaliophilite from Pollena (Vesuvius, Naples, Italy), with zone axis [001]. The reciprocal cell of kaliophilite is sketched in white, whereas the reciprocal vectors corresponding to the 15.6 Å periodicity are represented in gray. In (*b*) the corresponding HRTEM image is shown. The arrow indicates a slab with apparent 15.6 Å periodicity within the kaliophilite crystal. Other defects are also visible [modified from Bonaccorsi (1988 ▸)].

High-resolution transmission electron microscopy (HRTEM) with a [001] zone axis indeed reveals an oriented intergrowth of domains based on the *a* = 27 Å cell, separated by planes parallel to **c**, which may accumulate in places (Fig. 2 ▸) to simulate a smaller unit cell. The coherent domains with *a* = 27 Å cells are several hundred ångstroms wide. SAED patterns with a [001] zone axis indicated that the *hk*0 reflections with *h* − *k* = 3*n* were significantly stronger than those with *h* − *k* ≠ 3*n*, suggesting the occurrence of pseudo-translations with module *a*/3^1/2^. Differently oriented SAED patterns, however, did not point to a simple pseudo-cell with *a*′ = *a*/3^1/2^ and *c′* = *c*, because, in the *l* odd layers, the stronger reflections correspond to indices *h* − *k* ≠ 3*n* [see also Fig. 6(*a*) in the work of Cellai *et al.* (1992 ▸)].

### 3.3. *Ab initio* model obtained through three-dimensional electron diffraction   

The 3D ED technique (Kolb *et al.*, 2007 ▸; Rozhdestvenskaya *et al.*, 2017 ▸; Mugnaioli & Gemmi, 2018 ▸; Gemmi *et al.*, 2019 ▸) allowed for collecting sets of diffraction data from small single-crystal individuals with sizes of few hundreds of nanometres, namely the same size of the domains with *a* = 27 Å detected in HRTEM images (Fig. 2 ▸).

**Figure 3:**
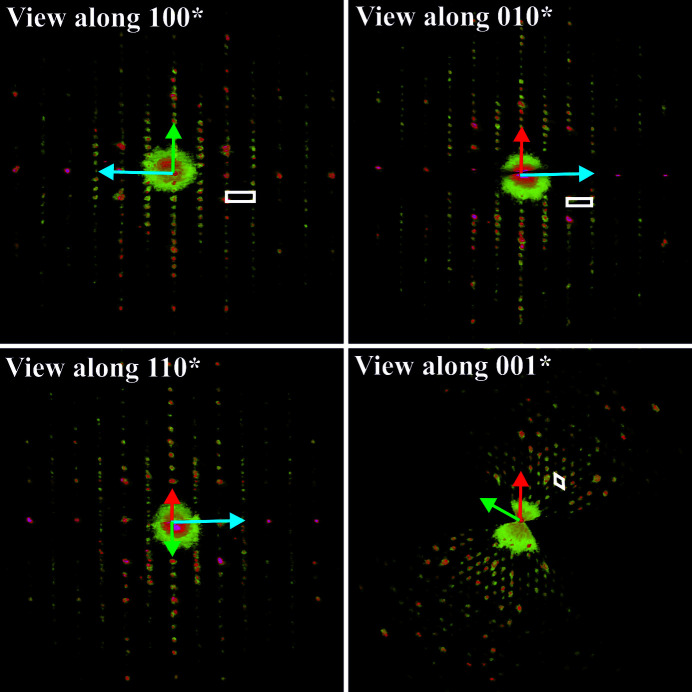
Projection of 3D ED reconstructed data taken from a single nanocrystal. (*a*) The view along 100*, (*b*) the view along 010*, (*c*) the view along 110* and (*d*) the view along 001*. Cell edges are sketched in white. Red arrows represent the projection of a* green arrows represent the projection of b* and blue arrows represent the projection of c*. Note that these are projections are along a 3D diffraction volume and are not conventional 2D oriented ED patterns.

The reconstructed 3D diffraction volume turned out to be consistent with a hexagonal unit cell with parameters *a* = 27.1 (5) and *c* = 8.6 (2) Å. Based on this cell, 11 743 experimental reflections were integrated, corresponding to the full reflection sphere with a resolution limit down to 1.0 Å (Fig. 3 ▸). The immediately striking feature of kaliophilite is that reflections *hkl* with *l* even and *h* − *k* = 3*n* are significantly stronger than those with *h* − *k* ≠ 3*n*. Conversely, reflections *hkl* with *l* odd and *h* − *k* = 3*n* are generally weaker than those with *h* − *k* ≠ 3*n*.

Moreover, reflections are absent or very weak for *h*0*l*: *l* = 2*n* + 1, which is possibly related to a *c*-glide plane in the structure. Still, because of the occurrence of minor dynamical effects that distribute intensity to the kinematically extinct reflections, it is rather controversial to assert, from ED, whether such an extinction rule is present or not. A structure solution was therefore attempted in several space groups belonging to either hexagonal or trigonal crystal systems.

A crystallochemically sound model was finally obtained in space group *P*3*c*1. All 11 K positions and all 18 Si/Al positions were found in the first Fourier map, together with 12 out of 36 O positions expected in order to have all Si and Al atoms in tetrahedral coordination. The crystallographic parameters are listed in Table S5.1. Remarkably, no solution was ever achieved assuming a hexagonal space group.

The remaining O atoms were inferred based on the tetrahedral coordination of Si and Al positions. A preliminary least-squares refinement already highlighted an alternating distribution of smaller and larger tetrahedra, suggesting a fully ordered arrangement of Si and Al.

### 3.4. Structure refinement by single-crystal X-ray diffraction   

**Figure 4:**
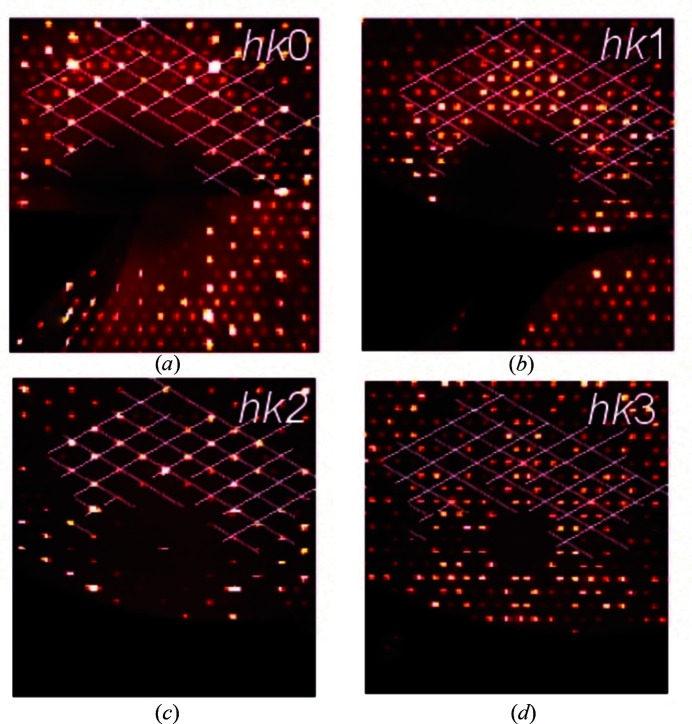
A scheme of the peculiar diffraction patterns in kaliophilite, based on the single-crystal X-ray diffraction experimental data. The four images show reconstructed sections of the reciprocal space, namely the planes (*a*) *hk*0, (*b*) *hk*1, (*c*) *hk*2 and (*d*) *hk*3. For comparison, the same grid is superimposed onto the four images, with nodes corresponding to reflections with *h* − *k* = 3*n*.

The trend observed in 3D ED intensities is confirmed by X-ray diffraction data. Reflections with *l* even are intense for *h* − *k* = 3*n* and weak for *h* − *k* ≠ 3*n*. Reflections with *l* odd are less intense throughout, but those with *h* − *k* = 3*n* are generally weaker than those with *h* − *k* ≠ 3*n*. This feature is easily observable in the reconstructed reciprocal-space sections (Fig. 4 ▸).

The X-ray diffraction data collected for the crystal from sample ms1 were used to refine the structural model. Refinement was performed with the program *SHELXL* (Sheldrick, 2015 ▸) starting in space group *P*3*c*1 and using the atomic coordinates of the model refined against 3D ED intensity data.

In the starting model, silicon and aluminium cations occupied alternating tetrahedral sites in agreement with the Loewenstein (1954 ▸) rule. However, refinement was unstable in this early stage and all T–O and O–O distances of the tetrahedral framework had to be restrained to maintain sound geometries. After several least-squares cycles, the reliability index could not be reduced below *R*1 = 0.180.

Taking into account the probable occurrence of pseudo-symmetries, which might have enhanced the observed symmetry of the X-ray and electron diffraction patterns and which are often responsible for oddities in the structure refinements, we lowered the symmetry from *P*3*c*1 to the subgroup *P*3. Note that the *P*3*c*1 > *P*3 group–subgroup descent is supported by the corresponding errors for intensity averaging in the Laue symmetries −3*m*1 (*R*_int_ = 0.095) and −3 (*R*_int_ = 0.064), the latter being indistinguishable from the uncertainty of the intensity data (*R*_sigma_ = 0.065).

To test the robustness of this model, no restraints were imposed on the T–O and O–O distances. The structural residual immediately dropped to *R*1 = 0.11 for isotropic displace­ment parameters, and after correcting for the occurrence of twinning with (1-10) as the twin plane, with twin fraction α = 0.45. In the successive refinement cycles, extra-framework sites 1 and 9B were found to host sodium cations (Na1 and Na9B, respectively) based on the observed short distances with the framework oxygen atoms and the site scattering power. Moreover, the extra-framework site 4B was split in two displaced sites, Na4B and K4B. The former has low occupancy and contains sodium cations which form four short bonds with the framework oxygen atoms (from 2.51 to 2.61 Å). The global K:Na ratio based on the refined electron density is 91:9, very close to the chemical data 90:10 (Table 2 ▸) for the ms1 kaliophilite. After anisotropic displacement parameters were introduced for the extra-framework cations, the residual dropped further to *R*1 = 0.077 for 33 165 reflections with *F*_o_ > 4σ(*F*_o_) and 0.090 for all 37 707 independent reflections.

Another possible twin plane is (2-10), which would invert the U and D directions of the tetrahedra as well as their occupation by Si and Al. A tentative refinement of the kaliophilite structure by introducing both twin planes was performed with the *JANA*2006 software (Petříček *et al.*, 2014 ▸), obtaining the following twin fractions: α_(1-10)_ = 0.27 and α_(2-10)_ = 0.15.

**Figure 5:**
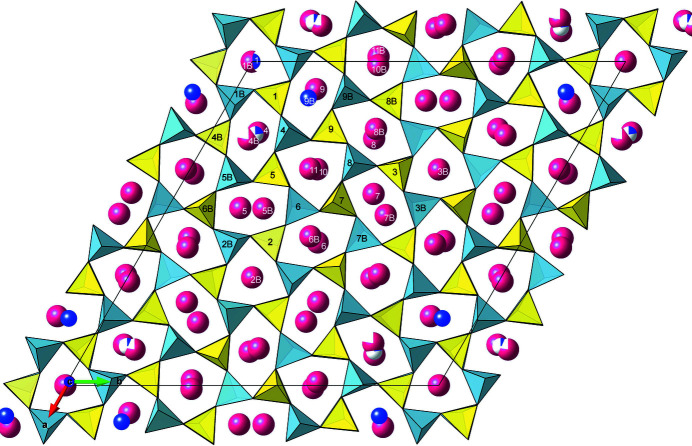
A unit cell of kaliophilite in space group *P*3, *a* = 27.06 and *c* = 8.56 Å. Si tetrahedra are shown in yellow, Al tetrahedra are shown in sky blue, Na cations are shown in blue and K cations are shown in red.

The refinement parameters are reported in Table S5.2. The final list of atomic coordinates and displacement parameters is available in Table S5.3, whereas selected bond distances for the framework and extra-framework cations are reported in Tables S5.4 and S5.5, respectively. The crystal structure of kaliophilite is represented in Fig. 5 ▸.

### 3.5. Rietveld refinements and microstructure   

Rietveld refinements were complex and are detailed in section S6 of the Supporting information. Immediately after the structure solution by 3D ED we tried to continue refinement in *P*3*c*1 using the Rietveld method but the residual never decreased below *R*(*F* ^2^) = 0.17. There were severe problems with anisotropic line broadening (ALB) along with pseudo-symmetry and reflection overlap which hindered convergence.

Using the X-ray single-crystal refined model, in space group *P*3 and with fixed atom parameters, convergence was reached at *R*(*F* ^2^) = 0.089, *R*(*F*) = 0.071 and χ^2^ = 117, confirming the correctness of the *P*3*c*1 > *P*3 group–subgroup reduction in symmetry without the possible bias of a twin law. The structural residuals compared well with their former counterparts obtained for twin crystal refinement against X-ray single-crystal data.

**Figure 6:**
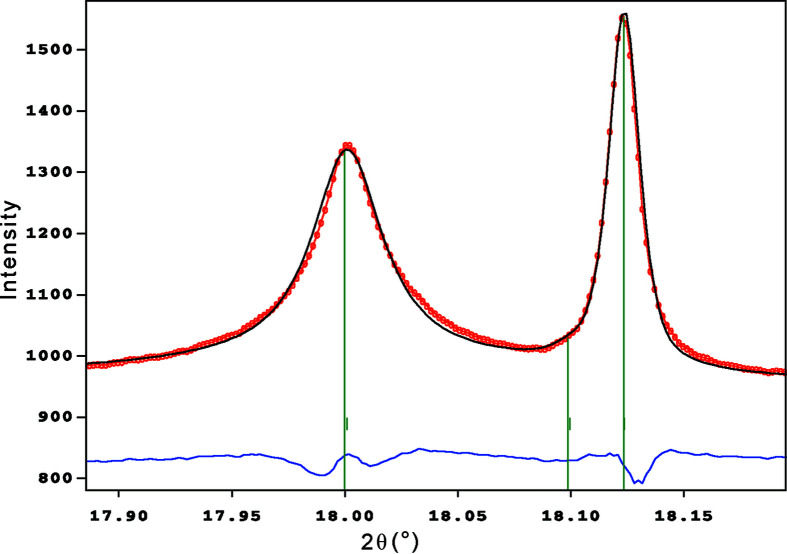
A Le Bail fit of reflections 511 (left) and 520 (right), showing the strong ALB with sharp *hkl*: *h* − *k* = 3*n* and broad *h − k* ≠ 3*n* lines. The calculated (black curve), observed (red dots) and difference patterns (blue curve below) are shown. Vertical lines indicate the Bragg positions. λ = 1.18091 Å.

A proper account of ALB was crucial for this result. Reflections complying with the *h* − *k* = 3*n* parity rule (*e.g.* 520 in Fig. 6 ▸) are sharp and approximately Gaussian, while the others are considerably broader and more Lorentzian, in agreement with the synchrotron single-crystal experiments (Fig. 1 ▸). To tackle the problem, line widths were made to obey two different profile functions and optimized by running a Le Bail refinement. Convergence was reached at χ^2^ = 43 and the obtained refined values for FWHM could be used to extract information about the domain size. Using integral breadths of the broad reflections, corrected for instrument and strain broadening, in the Scherrer equation, one obtains a rough estimate of 1100 Å for the kaliophilite coherent domains. This value refers to the domain size in the **ab** plane and compares rather well with results from HRTEM experiments.

The gap between the final Rietveld refinement and the Le Bail refinement (Δχ^2^ = 117 − 43 = 74) suggests that an improvement of atom parameters may be expected. However, an important discrepancy of about Δχ^2^ = 43 − 31 = 12 is also observed between the Le Bail refinement and the minimum (χ^2^ = 31) obtained when a super-Lorentzian shape is used to describe the broad peaks. This, together with grain-shape anisotropy and peak asymmetry, suggests that microstructure might explain the intensity mismatch more than structure itself. To test this hypothesis, we performed a first-principles energy minimization of the *P*3 structure using DFT. A small change in the atom coordinates and average energy (<0.46 pm and −4.1 µeV atom^−1^, respectively) was obtained, and the improved model showed almost ideal tetrahedral bond lengths of *d*(Si–O) = 1.63 (1) and *d*(Al–O) = 1.75 (1). Introducing this model in Rietveld calculations, the global and structural errors increased to χ^2^ = 146 and *R*(*F* ^2^) = 0.117. This clearly supports the microstructural origin of intensity mismatch, which is also likely to be the origin of the observed dispersion of T–O distances and atomic displacement parameters (ADPs).

Furthermore, it should be mentioned that the powder used for Rietveld refinement, though carefully selected and purified, turned out to contain a small amount (2.8%) of kalsilite. The association of kaliophilite and kalsilite was also observed by Aurisicchio & Federico (1985 ▸) in specimens from Alban Hills (Italy) and it is possible that the two phases have a common genesis.

Finally, the unit-cell dimensions obtained from Rietveld refinement for kaliophilite from sample cc1 are *a* = 27.0344 (2), *c* = 8.56362 (5) Å and *V* = 5420.28 (9) Å^3^, slightly different from those measured for the ms1 sample.

## 4. Description of the structure   

### 4.1. A new framework topology based on three different ring types   

The combination of 3D ED and X-ray diffraction data eventually allowed the determination of the structure of kaliophilite, based on a new and surprisingly complex framework of 36 independent TO_4_ tetrahedra alternatively occupied by Al and Si. This mineral crystallizes in space group *P*3 < *P*3*c*1 < *P-*6*c*2, where *P-*6*c*2 is the symmetry of the framework topology (the ‘aristotype’) after ignoring chemical alternation of Si and Al in the tetrahedra. This topology requires a unit cell of *a* = 27.06 and *c* = 8.56 Å, 27 times the cell of kalsilite, the simplest of the KAlSiO_4_ phases.

Kaliophilite is characterized by a new topology, and for a full appraisal of its complexity it is convenient to compare it with similar tetrahedral frameworks. Following Smith (1977 ▸), the frameworks with the tridymite topology and its variants are generated from a hexagonal tiling, *i.e.* 6^3^ nets (Schläfli symbol {6,3}), perpendicularly linked to the nodes of adjacent nets considering only ring-over-ring stacking. Links between consecutively stacked rings may occur at different places so that eight types of rings are possible (see Fig. S1.1).

In the family of tridymite and its variants there are now 13 different topologies (Table 3 ▸). Six of them (tri, BCT, ABW, CaG, BaF and par) are based on a single ring type, which is different for each topology. Six other topologies (WZP, ber, mal, ANZP, NZP, and O1) comprise two ring types. Finally, kaliophilite is the first example based on three ring types.

Table 3 ▸ lists for each topology the space group of the aristotype (or maximal symmetry, obtained ignoring topochemistry and conformational distortions) and the relative unit cell. To facilitate comparison, all space groups and unit cells are given in the setting where **c** is perpendicular to the six-membered rings, **a** is the period between adjacent rings and **b** is perpendicular to **a** in non-hexagonal space groups (*A* ≃ *a*_kalsilite_ = 5.16 Å, *B* ≃ 3^1/2^ *a*_kalsilite_ = 8.94 Å, *C* ≃ *c*_kalsilite_ = 8.70 Å). The different topologies impose differences in either unit-cell dimensions or space group. As an example, the unit cell 2*A B C* occurs in CaGa_2_O_4_ (CaG) and paracelsian (par), but the associated space groups are different. *Vice versa*, space group *P*6_3_/*mmc* occurs three times, in the tridymite (tri), WZP and malinkoite (mal) topologies, but with increasing unit-cell volumes, *i.e.* the density of symmetry elements per formula diminishes. This observation can be used to define a simple framework topology order parameter FTO = *k*/*t*_uc_, where *k* is the multiplicity of the general position of the aristotype space group and *t*_uc_ is the number of tetrahedra in the unit cell. FTO quantifies the complexity of the different topologies: the lower the FTO, the more complex the topology. The tridymite topology is evidently the simplest (FTO = 6), followed by BCT (FTO = 4) and ABW (FTO = 2). Kaliophilite has by far the most complex framework, with FTO = 1/9, *i.e.* 54 times lower than that for tridymite. Just for comparison, cristobalite would have FTO = 24, quartz would have 4, feldspar would have 1/2 and the complex zeolite MFI would have 1/12, only slightly smaller than kaliophilite.

The number of topologies based on more than one ring type is not limited as for topologies based on one ring type (Smith, 1977 ▸). With the discovery of a topology based on three different ring types, many more structures now seem possible.

### 4.2. Pseudo-symmetry and pseudo-extinctions   

**Figure 7:**
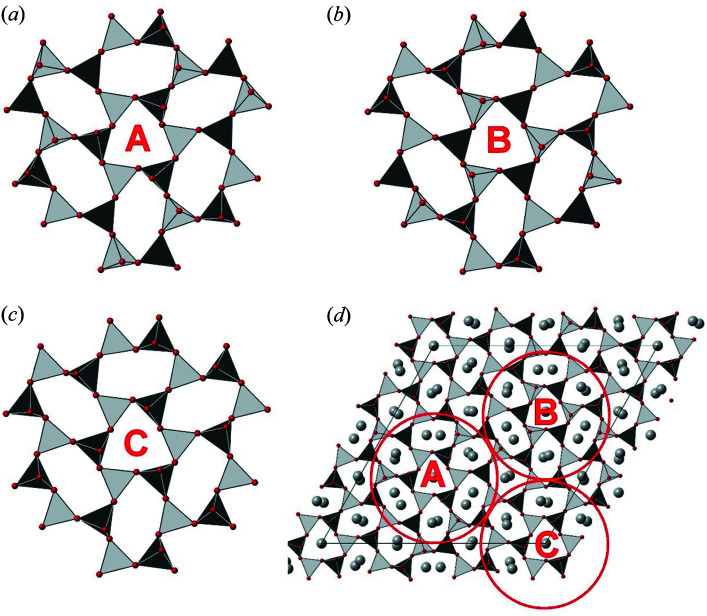
The crystal structure of kaliophilite as seen down **c**, with the three corollas indicated. (*a*), (*b*), (*c*) The three symmetry-independent corollas, A, B and C, occurring in kaliophilite are formed by a trigonal ring (1-3-5) at the center and six oval rings around it. The topologies of the oval rings are (1-2-4) and (1-2-3) in corollas A and B, whereas they are (1-3-5) in corolla C. Corollas A and B are related by the translation vector **t** = (1/3)**a** − (1/3)**b** + (1/2)**c**. (*d*) A drawing of the crystal structure of kaliophilite as seen down **c**, where the locations of the corollas, A, B and C, are emphasized by red circles.

It is convenient to look at the structure in terms of seven-ring units or ‘corollas’, similar to the characteristic motif known from nepheline (Fig. 7 ▸). Each corolla is formed by a central trigonal ring, surrounded by six oval rings. The structure of kaliophilite is made of three symmetrically independent corollas, each one centered on a different threefold axis. All of them have a trigonal (1-3-5) ring at the center whereas the oval rings around them have different topologies: (1-3-5) oval rings are around (0, 0, *z*), while alternated (1-2-3) and (1-2-4) oval rings are around (2/3, 1/3, *z*) and (1/3, 2/3, *z*) (Fig. 7 ▸). These corollas form a planar close packing, and the interstices correspond to (1-2-4) rings with approximately (non-crystallographic) trigonal shape.

Following the notation used in Fig. 7 ▸, it is worth noting that (i) the results of the structure refinement indicate that the K–Na substitution only occurs in corolla C around (0, 0, *z*), both in trigonal and oval (1-3-5) rings, and (ii) the A and B corollas are closely related to each other by a vector **t** = (1/3)**a** − (1/3)**b** + (1/2)**c**, by neglecting whether Si or Al occupy the tetrahedral sites.

The non-space-group translation relating corollas A and B implies that they very weakly contribute to reflections with odd *l* and *h* − *k* = 3*n*. This explains the observed enhancement of reflections with *h* − *k* ≠ 3*n* and odd *l*. Conversely, for reflections with even *l*, there is an enhancement of reflections with *h* − *k* = 3*n* (see section S7 in the Supporting information for a mathematical treatment).

### 4.3. Twinning by merohedry   

**Figure 8:**
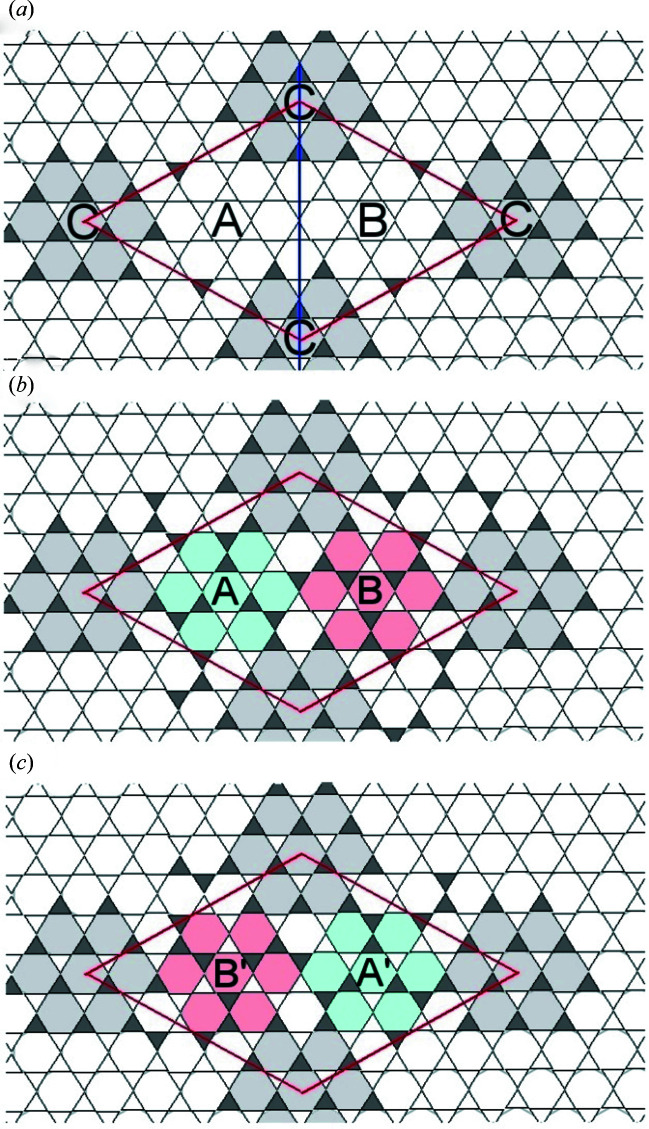
The topology of the 6^3^ net of kaliophilite; white and dark gray triangles represent tetrahedra pointing down and up, respectively. In all the images, (*a*), (*b*) and (*c*), the shaded hexagons (corolla C) correspond to rings where tetrahedra do not change their orientation in space after reflection in (1-10). In (*a*) the trace of one of the three mirror planes (1-10), valid only for corolla C, is indicated in blue. (*b*) The topology of kaliophilite with corolla A (in blue) and corolla B (in red), which are related to each other by the translation vector **t**_AB_ = (1/3)**a** − (1/3)**b** + (1/2)**c**. (*c*) The topology of the twin individual, with corolla A′ (in blue), which is obtained by reflecting A across (1-10), and corolla B′ (in red), which is obtained by reflecting B across (1-10).

A very useful way to visualize the framework topology of kaliophilite is given in Fig. 8 ▸. Considering only the topology of the framework, corolla C presents three mirror planes parallel to [001], which are symmetry operations that are not present in the *P*3 space group. It is possible to imagine the whole cell reflected on (1-10). In corolla C this is a symmetry element and all tetrahedra retain their U and D orientations. For corolla A, a mirror equivalent A′ [Figs. 8 ▸(*b*) and 8 ▸(*c*)] is created and superposed on corolla B, changing U/D in 22 out of 48 tetrahedra. This corresponds to the merohedric twin model used in the final model refined against single-crystal X-ray diffraction data.

Twin planes in kaliophilite apparently occur with a frequency of a few unit cells, as observed in HRTEM images (Fig. 2 ▸). A slight misorientation of twin domains may be the cause of the splitting of reflections observed in high-resolution single-crystal synchrotron diffraction (Fig. 1 ▸).

A second twinning mechanism appears to occur in the examined kaliophilite crystal, related to the twin plane (2-10). The effects of such twinning correspond to the inversion of the U and D orientations of all tetrahedra in the 6^3^ net, as well as to the inversion of the Si and Al occupancy of tetrahedra. This additional merohedric twinning may be the actual cause of the observed average T–O distances in Si- and Al-centered tetrahedra, which mimic partial Si/Al disorder in kaliophilite.

A statistical occurrence of both twin operators, namely the twin planes (1-10) and (2-10), combined with the Friedel law, could explain the observed 6/*mmm* Laue symmetry reported in the past for many kaliophilite samples.

Another interesting observation refers to the *h* − *k* = 3*n* parity rule and the width of reflections. Reflections with *h* − *k* ≠ 3*n* are significantly broader when compared with the very sharp reflections with *h* − *k* = 3*n*, as observed in both powder and single-crystal synchrotron diffraction. Ignoring the Al/Si atoms and the apical oxygens, which require the 27 Å cell, the continuous layer of the basal oxygen atoms and most K ions roughly correspond to the sub-cell *a*′ = *a*/3^1/2^ = 15.6 Å (Fig. 5 ▸). With the twin law (2-10), this sub-cell would extend over both individuals, such as to enlarge the coherent domains in **ab**.

Finally, it is also important to point out that occurrences of micro-twinning and pseudo-symmetries are both related to the geometries of corollas but affect kaliophilite diffraction data in two different ways, which are difficult to deconvolute. The small size of merohedric twin domains may account for the relatively broad reflections corresponding to the 27 Å periodicity (*hkl*: *h* − *k* ≠ 3*n*), as observed in powder and single-crystal synchrotron data. The occurrence of a non-space-group translation is responsible for pseudo-extinctions that make reflections *h* − *k* = 3*n* very strong in layers with even *l* and weak in layers with odd *l*. This model is able to describe all experimental results in a satisfactory way, but we are aware that the quantitative solution of the microstructure still needs more experimental work.

### 4.4. Distances and angles within the [AlSiO_4_] framework   

The grand mean of (Al,Si)–O distances, 〈〈T–O〉〉 = 1.68 (3) Å (Table S5.4), is indistinguishable from the expected value (1.682 Å; Jones, 1968 ▸) for an Al:Si = 1:1 composition and agrees with the results from elemental analysis and ^29^Si MAS–NMR spectroscopy. The mean distances for Si–O and Al–O are 1.652 (9) and 1.713 (8) Å, respectively, closer to one another than expected for full Al/Si order. This suggests that long-range order might be affected by microstructural features, which are not simulated in the present model.

The most visible effect of the *P*3*c*1 > *P*3 group–subgroup change is a slight rotation of the six-membered rings centered on the threefold axes, which distorts the six surrounding oval rings (the ‘corolla’ described above). The corolla remains eclipsed as seen along [001], but tetrahedra tilt so that axial Al–O–Si angles are more bent than in the *P*3*c*1 model (152° instead of 162°), and hence are more similar to the mean of the equatorial Al–O–Si angles (140°) and to the expected value (142°; Liebau, 1985 ▸). Remarkably, this tiny adjustment of positions, governed by the correct symmetry relaxation and collectively applied to many atoms, allowed convergence of the refinement to a minimum in all residuals.

Lippmaa *et al.* (1986 ▸) found that the chemical shift of tetrahedral aluminium in ^27^Al NMR spectra is linearly correlated with the Al–O–Si angle in crystalline aluminosilicate minerals and zeolites. A more recent empirical relationship between the isotropic chemical shift and the Al–O–Si angle is proposed in the work of Angeli *et al.* (2000 ▸):
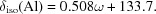
For kaliophilite, δ_iso_(Al) = 60.3 gives a calculated angle ω of 144.5°, which matches quite well with the average refined angle of 143° in the *P*3 space group.

### 4.5. Ring conformation   

**Figure 9:**
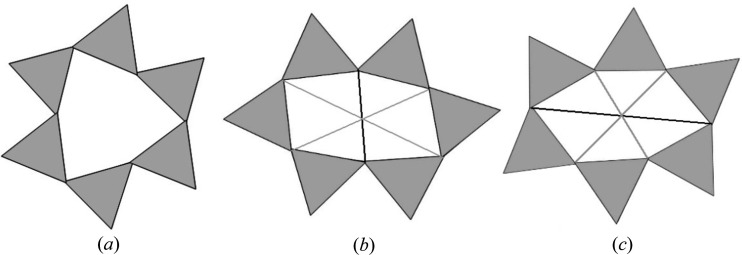
Different D6R conformations observed in kaliophilite. (*a*) Eclipsed trigonal (*et*), (*b*) eclipsed oval oblate (*eo*) and (*c*) eclipsed oval prolate (*ep*).

A convenient way to describe the ring conformation is based on two features: the tetrahedral bases may stack either eclipsed (*e*) or staggered (*s*) along **c** to form a double six-ring cage (D6R in zeolite chemistry). Moreover, the rings may have hexagonal (*h*), trigonal (*t*) or oblate and prolate oval [(*o*), (*p*)] appearance. In kaliophilite, all the double rings are eclipsed (*e*) and three conformations coexist, which may be conveniently named as *et, eo* and *ep* (Fig. 9 ▸). The *et* conformation is known from trigonal kalsilite, and the *eo* conformation is known from nepheline, trikalsilite and panunzite. The *ep* conformation is a prolate oval, not clearly observed before in K[AlSiO_4_] compositions.

The definition of oblate (*o*) and prolate (*p*) refers to two basal oxygens in *para* position which form either the short (*eo*) or long (*ep*) axis of an ellipsoid [thick lines in Figs. 9 ▸(*b*) and 9 ▸(*c*)], while the two remaining *para* positions show more similar distances [thin lines in Figs. 9 ▸(*b*) and 9 ▸(*c*)]. With ∼6.77 (5) *versus* 2 × 4.45 (40) Å in the (*ep*) rings and 3.80 (9) *versus* 2 × 5.97 (36) Å in the (*eo*) rings, all distances are well apart from the grand mean at ∼5.24 (1.13) Å.

### 4.6. Extra-framework cation coordination and electrostatic valence balance   

Extra-framework cations are trapped between pairs of rings belonging to adjacent sheets in 22 symmetry-independent sites and their coordination depends both on the topology and conformation of such rings. As seen down **c**, the extra-framework cations are located approximately in the center of the (1-3-5) and (1-2-4) double rings (Fig. 5 ▸), whereas they are neatly displaced from the center in the (1-2-3) double rings. As a consequence of the different ring conformations, *et, eo* and *ep*, the coordination polyhedra of the extra-framework cations are quite different, ranging from tricapped triangular prisms to six, seven, eight, nine and tenfold asymmetric polyhedra, with bond distances ranging from 2.5 to 3.4 Å (Table S5.5). In the Na1, Na4B and Na9B sites, all located in corolla C around (0, 0, *z*), the short bond distances indicate a complete or partial substitution of potassium by sodium cations, confirmed by the refinement of the site scattering power.

Bond-valence analysis was performed for all atoms located in the crystal structure of kaliophilite, using the parameters of Brese & O’Keeffe (1991 ▸) for the Si–O, Al–O, K–O and Na–O bonds. The average bond-valence sum (BVS) is 2.01 (7) v.u. for oxygen atoms, 3.72 (9) v.u. for Si, 3.39 (7) v.u. for Al (these values are in agreement with the apparent partial Si–Al disorder in the tetrahedral sites) and 0.9 (3) v.u. for the extra-framework cations.

## 5. Conclusions   

The structure determination of kaliophilite remained an open question for more than a century, crossing the whole history of X-ray crystallography, despite the simple chemical composition of the mineral and its apparently well resolved diffraction pattern. The failure of previous approaches was caused by the occurrence of pseudo-symmetry and micro-twinning, which could only be unveiled by combining different experimental techniques. Thanks to the recent advent of 3D ED, it was eventually possible to get single-crystal data from sub-micrometric, non-twinned and almost defect-free domains of kaliophilite, from which a first model could be obtained *ab* *initio*. The key aspect revealed by 3D ED was the trigonal symmetry, while all attempts in the past were focused on the presence of a sixfold symmetry axis. The structural model was refined by single-crystal X-ray data using a subgroup symmetry and after imposing a proper twin model. HRTEM imaging and accurate peak-shape analysis by synchrotron radiation allowed understanding of the twinning and microstructure mechanisms.

Despite its simple chemical composition, kaliophilite enshrines a very complex structure, as measurable by information-based complexity parameters (Krivovichev, 2017 ▸). The information content per atom, *I*_G_ = 7.002 bits atom^−1^, and the total information content, *I*_G, total_ = 2646.922 bits unit cell^−1^ (calculated with *ToposPro* 5.4.0.2; Blatov *et al.*, 2014 ▸) are indeed much higher than the values reported for kalsilite (*I*_G_ = 1.896 bits atom^−1^ and *I*_G, total_ = 45.510 bits unit cell^−1^ for kalsilite 1H, and *I*_G_ = 2.128 bits atom^−1^ and *I*_G, total_ = 29.793 bits unit cell^−1^ for kalsilite 1T) and for its known complex modification panunzite (*I*_G_ = 5.279 bits atom^−1^ and *I*_G, total_ = 1182.496 bits unit cell^−1^). The *I*_G, total_ value would rank kaliophilite among the 20 most complex minerals known to date (Krivovichev, 2013 ▸).

Among the known phases with tridymite topology and its variants, kaliophilite is the only one that contains three different types of six-membered rings, namely (1-3-5), (1-2-4) and (1-2-3). After this work, the number of known different framework topologies in the family increases to 13, seven of which are based on more than one ring type.

The number of structural combinations that can be obtained by mixing more than one type of ring is only limited by the size of the unit cell, but the existence of kaliophilite indicates that this size might actually be quite respectable. This opens up new perspectives to many more topologies, and new channel systems can now be conceived.

## 6. Related literature   

The following references are cited in the Supporting information for this article: Abbott (1984) ▸; David (2004) ▸; Gozzo *et al.* (2010) ▸; Gregorkiewitz *et al.* (1991) ▸; Herbstein (2000) ▸; Larson & Von Dreele (2004) ▸; Mügge (1927) ▸; Sahama (1962) ▸; Scacchi (1888) ▸; Sobrados (1991) ▸; Stebbins *et al.* (1986) ▸; Stephens (1999) ▸; Tian & Billinge (2011) ▸; Zambonini (1910) ▸.

## Supplementary Material

Crystal structure: contains datablock(s) I. DOI: 10.1107/S2052252520012270/fc5047sup1.cif

Supporting information, figures and tables. DOI: 10.1107/S2052252520012270/fc5047sup2.pdf

CCDC reference: 2027405

## Figures and Tables

**Table 1:** The 12 known phases with compositions close to KAlSiO_4_ and based on tridymite topology and its variants, along with nepheline (Na,K)[AlSiO_4_] K/2T = [K/(Al + Na)], cell parameters and ring topologies are reported for each phase. Formula volume *V*_m_ and cell content *Z* refer to (K,Na)AlSiO_4_. In the ‘Symmetry’ column, the space group of the structure is indicated, whenever it is known; otherwise, any available symmetry information (lattice, Laue class, diffraction symbol) is given. Synthetic phases are underlined.

Phase	K/2T	*a* (Å)	*b* (Å)	*c* (Å)	β (°)	*V*_m_ (Å^3^)	*Z*	Symmetry	Ring topologies
Kalsilite (Perrotta & Smith, 1965 ▸)	0.98	5.16	5.16	8.69	–	100.3	2	*P*6_3_	(1-3-5)
Trigonal kalsilite (Cellai *et al.*, 1997 ▸)	0.997	5.16	5.16	8.71	–	100.3	2	*P*31*c*	(1-3-5)
Nepheline (Tait *et al.*, 2003 ▸)	0.24	10.00	10.00	8.38	–	90.75	8	*P*6_3_	(1-3-5) × 4
Trikalsilite (Bonaccorsi *et al.*, 1988 ▸)	0.67	15.34	15.34	8.50	–	96.2	18	*P*6_3_	(1-3-5) × 9
Panunzite (Merlino *et al.*, 1985 ▸), *H*4	0.72	20.50	20.50	8.55	–	97.2	32	*P*6_3_	(1-3-5) × 16
Megakalsilite (Khomyakov *et al.*, 2002 ▸)	0.999	18.11	18.11	8.46	–	100.2	24	*P*6_3_	(1-3-5) × 3 (1-2-3) × 9
ABW † (Minor *et al.*, 1978 ▸)	∼1	10.55	18.15	8.49	–	101.6	16	*mmm, C*-*c*-	(1-2-3) × 8
*O* 1 (Gregorkiewitz, 1980 ▸)	1	15.67	9.07	8.56	90.13	101.4	12	*P*2_1_	(1-2-3) × 2 (1-2-4) × 4
*O* 2 (Smith & Tuttle, 1957 ▸)	0.85	10.47	8.89	8.55	–	99.4	8	o*P*	Unknown
*H* 2 (Smith & Tuttle, 1957 ▸)	1	5.18	5.18	8.56	–	99.4	2	h*P*	Unknown
*O* 1-hT (Cook *et al.*, 1977 ▸)	1	15.60	18.11	8.56	–	100.8	24	*mmm, Pb*--	Unknown
BCT † (Cook *et al.*, 1977 ▸; Dollase & Ross, 1993 ▸)	1.1	5.22	8.94	8.94	–	104.4	4	4/*mmm, I*----	(1-4) × 2
Kaliophilite (this work)	0.91	27.06	27.06	8.56	–	100.5	54	*P*3	(1-3-5) × 9 (1-2-3) × 6 (1-2-4) × 12

† ABW and BCT are iza codes (https://europe.iza-structure.org) for the respective framework topologies. The ‘tetragonal phase’ reported by Cook *et al.* (1977 ▸) is Na free and has an alkali excess of ∼K_1.1_Al_1.1_Si_0.9_O_4_.

**Table 2:** Results of elemental analyses obtained for several crystals of kaliophilite from Colle Cimino and Monte Somma *x* represents the number of points analyzed and used for averaging and *y* represents the number of analyzed grains. ‘Nd’ stands for not detected. Analyses of sample cc2 were performed on nanometric fragments using a transmission electron microscope and have a dispersion not comparable with other measurements.

Sample	Colle Cimino cc1	Colle Cimino cc2	Colle Cimino cc3	Pollena ms1	Monte Somma ms2
*x, y*	3, 3	2, 1	30, 3	15, 1	30, 3
K_2_O	28.13 (14)	26.04	29.41 (24)	27.42 (23)	28.17 (23)
Na_2_O	0.98 (50)	1.19	0.68 (8)	2.03 (9)	1.69 (10)
CaO	Nd	Nd	0.00	0.01 (1)	0.00
Fe_2_O_3_	1.34 (15)	1.36	0.04 (3)†	0.04 (2)	0.04 (3)
Al_2_O_3_	30.77 (48)	32.28	32.20 (21)	31.65 (49)	32.63 (33)
SiO_2_	36.91 (76)	39.13	38.93 (36)	39.70 (30)	38.55 (34)
Total	98.13	100.00	101.26	100.78	101.08
Elements, based on four oxygen atoms per formula unit					
K	0.966 (5)	0.88	0.973 (16)	0.901 (8)	0.931 (9)
Na	0.051 (26)	0.06	0.034 (12)	0.102 (5)	0.085 (5)
Ca	–	–	0	0.02	0
Fe	0.027 (3)	0.03	0.001 (1)	0.001 (1)	0.001 (1)
Al	0.976 (15)	1.00	0.984 (9)	0.964 (10)	0.996 (7)
Si	0.993 (20)	1.03	1.010 (8)	1.026 (8)	0.999 (6)

† FeO.

**Table 3:** The 13 known frameworks of the topological family of tridymite and its variants The acronym is underlined if it is realized in the composition of KAlSiO_4_. See the main text for explanation of all the parameters. Tri = SiO_2_ (tridymite-hT; Gibbs, 1926 ▸), BCT = Ca[AlSiO_4_]_2_ [Takéuchi *et al.* (1973 ▸); the natural occurrence as mineral svyatoslavite was later reported by Krivovichev *et al.* (2012 ▸)], ABW = (N_2_H_5_)LiSO_4_ (Brown, 1964 ▸), CaG = CaGa_2_O_4_ (Deiseroth & Müller-Buschbaum, 1973 ▸), BaF = BaFe_2_O_4_ (Mitsuda *et al.*, 1971 ▸), par = Ba[AlSiO_4_]_2_ (paracelsian; Smith, 1953 ▸), WZP = (H_3_O)ZnPO_4_ (Sandomirskii *et al.*, 1977 ▸), ber = NaBePO_4_ (beryllonite; Golovastikov, 1962 ▸), mal = NaBSiO4 (malinkoite; Sokolova *et al.*, 2001 ▸), ANZP = (NH_4_)Na_3_[ZnPO_4_]_4_ (Harrison, 2000 ▸), NZP = (Na,K)ZnPO_4_ (Yakubovich & Melnikov, 1989 ▸), O1 = KAlSiO_4_ *O*1 (Gregorkiewitz, 1980 ▸) and Kp = kaliophilite (this work).

Acronym	Space group	Unit cell	*t*_uc_	FTO	Ring topologies
Tri	*P*6_3_/*mmc*	A, C	4	6	(1-3-5)
BCT	*I*4/*mmm*	B, A	8	4	(1-4)
ABW	*Imam*	A, B, C	8	2	(1-2-3)
CaG	*Bbcm*	2A, B, C	16	1	(1-2-4)
BaF	*Amam*	A, 2B, C	16	1	(1)
Par	*Bbmm*	2A, B, C	16	1	(1-2)
WZP	*P*6_3_/*mmc*	2A, C	16	3/2	(1-2-3), (1-3-5)
Ber	*Imam*	3A, B, C	24	2/3	(1-2-4), (1-3-5)
Mal	*P*6_3_/*mmc*	3A, C	36	2/3	(1-2-4), (1-3-5)
ANZP	*Bbmm*	2A, 2B, C	32	1/2	(1-2), (1-2-3)
NZP	*P*6_3_/*mcm*	2B, C	48	1/2	(1-2-4), (1-3-5)
O1	*Pnam*	3A, B, C	24	1/3	(1-2-3), (1-2-4)
Kp	*P*-6*c*2	3B, C	108	1/9	(1-2-3), (1-2-4), (1-3-5)
