# Supplementary material for: The structure of kaliophilite KAlSiO4, a long-lasting crystallographic problem
Source: IUCrJ. 2020 Sep 29;7(Pt 6):1070–83. doi: 10.1107/S2052252520012270 (PMC7642771; doi:10.1107/S2052252520012270)
Supplement: Supplementary file 2 [file m-07-01070-sup2.pdf]

IUCrJ

Volume 7 (2020)

Supporting information for article:

The structure of kaliophilite KAlSiO4, a long-lasting crystallographic problem

Enrico Mugnaioli, Elena Bonaccorsi, Arianna E. Lanza, Erik Elkaim, Virginia Diez-Gómez, Isabel Sobrados, Mauro Gemmi and Miguel Gregorkiewitz

The structure of kaliophilite $KAlSiO_4$, a long-lasting crystallographic problem

Enrico Mugnaioli[a], Elena Bonaccorsi[b]*, Arianna E. Lanza[a], Erik Elkaim[c], Virginia Diez-Gómez[d], Isabel Sobrados[d], Mauro Gemmi[a]*, Miguel Gregorkiewitz[e]*

[a]Center for Nanotechnology Innovation@NEST, Istituto Italiano di Tecnologia, Piazza S. Silvestro 12, Pisa, 56127, Italy

[b]Dipartimento di Scienze della Terra, Università di Pisa, Via Santa Maria 53, Pisa, 56126, Italy

[c]Synchrotron Soleil, L'Orme des Merisiers, Saint-Aubin, Gif-sur-Yvette, 91192, France

[d]Instituto de Ciencia de Materiales de Madrid, Consejo Superior de Investigaciones Científicas (ICMM-CSIC), Sor Juana Inés de la Cruz 3, Madrid, 28049, Spain

[e]Dipartimento di Scienze Fisiche, della Terra e dell'Ambiente, Università di Siena, Via Laterina 8, Siena, 53100, Italy

Correspondence email: elena.bonaccorsi@unipi.it; mauro.gemmi@iit.it; gregormigu@gmail.com

Supporting Information:

S1. Ring types in 6^3 nets; chemical composition

S2. ^{29}Si and ^{27}Al MAS-NMR spectra

S3. Optical microscopy and Weissenberg X-ray diffraction patterns

S4. High resolution synchrotron single crystal diffraction scans

S5. Crystal data, atom parameters, T-O and K-O interatomic distances

S6. Rietveld refinements using high resolution synchrotron radiation

S7. Pseudo-extinction rule

S1. Ring types in 6^3 nets; chemical composition

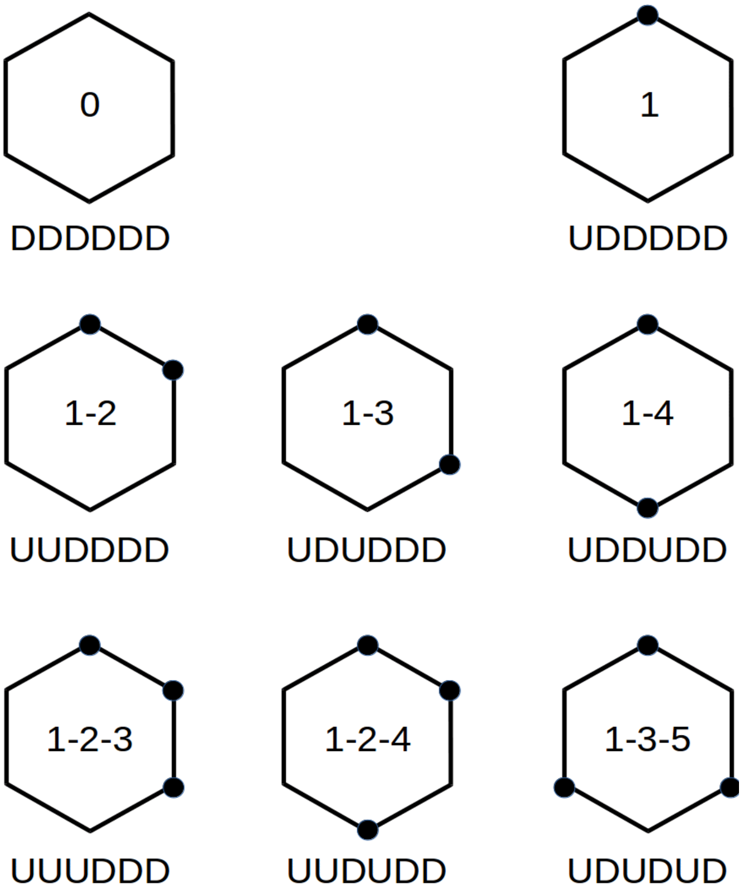

Figure S1.1. Possible types of six-membered rings of tetrahedral nodes. In a 6^3 net, all nodes are linked to three neighbours in the basal plane. Dots stand for nodes where the apical vertex points upwards (U), the remainder points downwards (D).

Table S1.1. Published chemical analyses, densities and optical constants of kaliophilite (wet analyses except the last five). All values from original paper except sca88 and zam10 for which 2 secondary references (mug27 and ban31) have been used. All elements have been recalculated from oxides per 4 oxygens.

**Locality**	Vesuv	Somma	Vesuv	Vesuv	Somma	Vesuv	Vesuv	Somma	CC	CC	Ariccia	Somma	CC	Vesuv	MF	MF
**Oxides (mass%)**																
SiO2	40.43	37.45	37.73	38.53	39.04	38.0	39.2	38.62	38.17	38.73	39.05	38.87	39.41	38.73	38.0	38.2
Al2O3	33.07	32.43	33.09	32.20	31.96	28.8	33.36	32.09	31.73	31.04	30.31	31.89	32.75	31.55	30.2	31.3
Fe2O3	-	-	-	0.12	0.98	-	-	0.80	0.18	tr	tr	-	-	-	2.22	0.77
FeO	-	-	-	-	-	-	-	0.29	0	0	0	0.07	0.34	0.21	0	0
MgO	-	-	-	tr	0.15	-	-	0	tr	0	0	-	0	0	0	0
CaO	3.07	2.18	-	0.28	0.33	0.5	0.47	0	1.87	2.39	1.09	0.05	0	0	0	0.11
Na2O	-	2.26	0.37	2.12	3.89	tr	2.88	3.77	2.17	0.77	1.68	0.77	3.26	1.04	1.28	1.35
K2O	22.25	27.20	29.30	26.62	22.84	32.2	24.13	24.18	24.56	23.97	25.75	28.76	24.07	28.07	27.3	27.7
H2O	1.18	-		-	0.60	-	-	0.23	1.23	2.98	1.88	-	-	-	-	-
total	100.00	101.52	100.49	100.16[1]	99.79	99.5	100.04	99.98	99.95[2]	99.96[3]	99.80	100.41	99.83	99.6	99.00	99.62[4]
**Elements per 4 oxygens**																
Si	1.03	0.97	0.99	1.00	1.01	1.03	1.00	1.00	1.00	1.03	1.04	1.015	1.01	1.02	1.01	1.01
Al	0.99	0.99	1.02	0.99	0.98	0.92	1.01	0.98	0.98	0.97	0.95	0.981	0.99	0.98	0.94	0.97
Fe3+	-	-	-	tr	0.02	-	-	0.02	tr	tr	tr	-	-		0.04	0.02
Fe2+	-	-	-	-	-	-	-	0.01	0	0	0	0.001	0.01	0.01	0	0
Ca	0.08	0.06	-	0.01	0.01	0.01	0.01	0	0.05	0.07	0.03	0.002	0	0	0	0
Na	-	0.11	0.02	0.11	0.20	tr	0.14	0.19	0.11	0,04	0,09	0.039	0.16	0.05	0.07	0.07
K	0.72	0.90	0.98	0.89	0.75	1.11	0.79	0.80	0.82	0.81	0.87	0.956	0.79	0.94	0.92	0.93
**Density (g/cm^3)**																
ρ	2.67	2.602	2.493	2.628	2.61	2.598	2.606									
**Optical properties**																
ε					1.5273	1.527	1.5258	1.5303	1.5312	1.5289	1.5294					
ω					1.5316	1.531	1.5296	1.5345	1.5362	1.5338	1.5349					
Δn = ε-ω		<0			-0.0043	-0.004	-0.0038	-0.0042	-0.0050	-0.0049	-0.0055					
**Reference**	cov39	mie87	sca88	zam10	mug27	ban31	ban31	sah62[5]	bar70	bar70	bar70	abb84	aur85	cel92	dib01	dib01

Abbreviated localities: Vesuv = Vesuvius, CC = Colle Cimino, MF = Montefiascone. - stays for not determined; 0 stays for below detection limit / not detected; tr stays for traces; [1]: contains 0.14% Cl, [2]: contains 0.04% Rb_2O, [3]: contains 0.08% Rb_2O; [4]: contains traces of Mn; [5]: optical properties determined by bar70.

References. abb84: Abbott (1984); aur85: Aurisicchio & Federico (1985); ban31: Bannister & Hey (1931); bar70: Barbieri *et al.* (1970); cel92: Cellai *et al.* (1992); cov39: Covelli (1839); dib01: Di Battistini *et al.* (2001); mie87: Mierisch (1887); mug27: Mügge (1927); sah62: Sahama (1962); sca88: Scacchi (1888); zam10: Zambonini (1910).

References

Abbott Jr., R. N. (1984). *Am. Mineral.* **69**, 449–457.

Aurisicchio, C. & Federico, M. (1985). *Bull. Geol. Soc. Finland* **57**, 129–137.

Bannister, F. A. & Hey, M. H. (1931). *Mineral. Mag.* **22**, 569–608.

Barbieri, M., Federico, M. & Tolomeo, L. (1970). *Period. Miner.* **39**, 323–341 (in Italian)

Cellai, D., Carpenter, M. A. & Heaney, P. J. (1992). *Eur. J. Mineral.* **4**, 1209–1220.

Covelli, N. (1839). *Atti Acc. Sc. Napoli* **4**, 17–32 (in Italian).

Di Battistini, G., Montanini, A., Vernia, L., Venturelli, G. & Tonarini, S. (2001). *Lithos* **59**, 1–24.

Mierisch, B. (1887). *Tschermak's Miner. Petr. Mittheil.* **8**, 113−189 (in German).

Mügge, O. (1927). *Z. Kristallogr.* **65**, 381−390 (in German).

Sahama, T. G. (1962). *Norsk Geol. Tidsskrift* **42**, 168−179.

Scacchi, E. (1888). *Rend. Accad. Fis. Mat. Napoli ser. 2*, **2**, 487 (in Italian).

Zambonini, F. (1910). *Rend. Accad. Fis. Mat. Napoli* **49**, 83 (in Italian).

S2. ^{29}Si and ^{27}Al MAS-NMR spectra

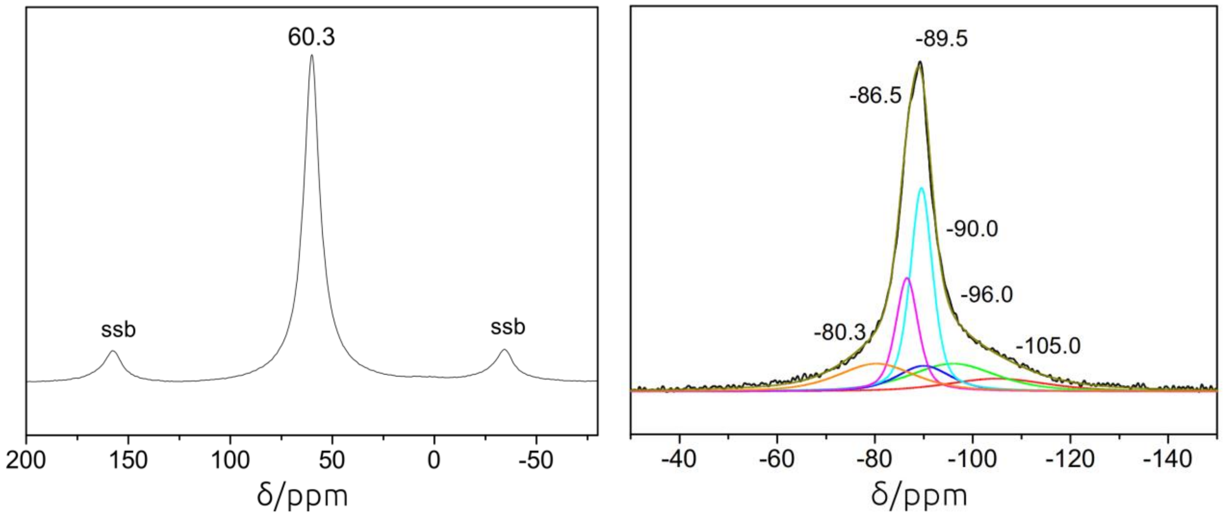

Figure S2.1. ^{27}Al (left) and ^{29}Si MAS-NMR (right) spectra of kaliophilite from Colle Cimino (sample cc1); black observed, yellow sum of contributions. ssb indicates spinning sidebands.

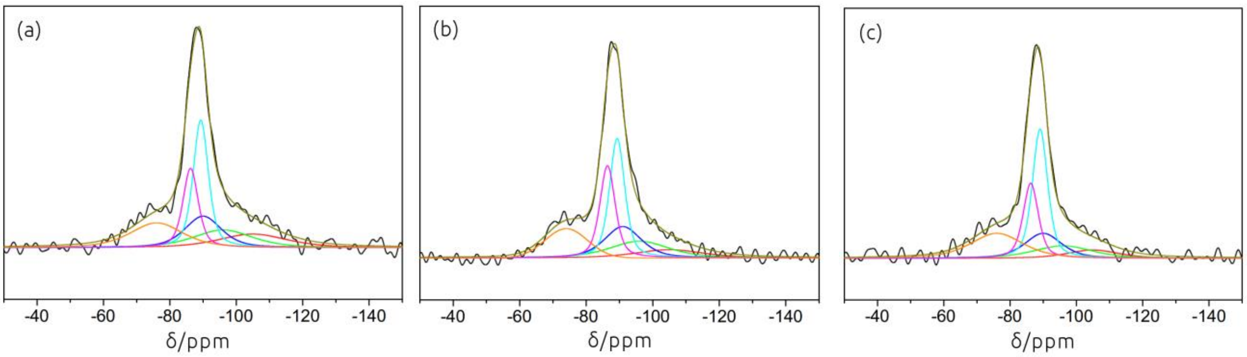

Figure S2.2. ^{29}Si MAS-NMR spectra of kaliophilite from three different samples from Colle Cimino: #C1 (a), #CL (b) and #IH (c).

^{29}Si MAS-NMR spectra were obtained for various kaliophilite samples from Colle Cimino, using exceptionally long accumulation times to allow interpretation of the broad base of the peaks. The spectra of the four samples in Fig. S2.1 and Fig. S2.2 show clearly that there is a difference in the left, but not in the right part of the base. We therefore concluded that the left part is probably due to some impurity which changes from sample to sample, while the right part is inherent to kaliophilite itself.

To confirm the hypothesis, we simulated a spectrum containing six components: a central doublet due to kaliophilite (δ = -86.5 and 89.5 ppm), and four broad peaks representing an impurity at about -74 to -80 ppm, a sum of distorted kaliophilite Q^4(4Al) environments at around -90 ppm, and two environments resulting from Si for Al substitution in kaliophilite, *i.e.* Q^4(3Al) at about -96 ppm and Q^4(0Al) at about -105 ppm. The latter two have been found earlier for Bancroft nepheline and its K-exchanged derivative (Stebbins *et al.*, 1986; Sobrados, 1991) and are thought to be associated with alkali vacancies and twin boundaries. All peaks were found from curve fitting with some restriction for the -96 and -105 ppm positions. Fitting results are summarized in Table S2.1.

The central doublet was also observed in the ^{29}Si MAS-NMR spectrum of *O*1 and the amount of disordered Q^4(4Al) and Q^4(3Al) and Q^4(0Al) environments in kaliophilite is compatible with earlier findings for nepheline and *O*1 (Stebbins *et al.*, 1986; Gregorkiewitz *et al.*, 2008). The amount of impurities, on the other hand, seems

high and this is not supported by powder diffraction where crystalline impurities can be excluded at a level of about 0.4%, and amorphous impurities seem improbable.

Table S2.1. Chemical shifts δ, widths and areas as obtained from fitting of the six ^{29}Si peaks in Fig. S2.1 and Fig. S2.2. Samples are cc1 (1st entry), #C1 (2nd entry), #CL (3rd entry) and #IH (4th entry). Kp stands for kaliophilite.

	"impurity"	Kp Q^4(4Al)		Kp $Q^4(4Al)_{dis}$	Kp Q^4(3Al)	Kp Q^4(0Al)
δ/ppm	-80<br>-76<br>-74<br>-76	-86.5<br>-86.2<br>-86.4<br>-86.2	-89.5<br>-89.3<br>-89.3<br>-89.1	-90<br>-90<br>-91<br>-90	-96<br>-96<br>-96<br>-96	-105<br>-105<br>-105<br>-105
FWHM/ppm	19<br>19<br>15<br>19	5.4<br>5.4<br>5.4<br>5.4	5.4<br>5.4<br>5.4<br>5.4	13<br>13<br>13<br>13	21<br>21<br>21<br>21	24<br>24<br>24<br>18
area/%	14.7<br>16.9<br>14.8<br>19.8	18.1<br>16.4<br>20.6<br>17.9	32.5<br>26.3<br>26.6<br>30.9	9.7<br>15.2<br>16.5<br>14.1	16.4<br>13.4<br>13.8<br>11.0	8.6<br>11.8<br>7.7<br>6.4

The central kaliophilite doublet (Fig. S2.3) was calculated *ab initio* using CASTEP (Clark *et al.*, 2005) for the model given in Table S5.3, which contains 18 crystallographically different Si positions, space group *P*3. While the positions are very similar to the fitting results, intensities appear inverted with the higher peak at about -86.4 ppm. The nuclei probed in NMR are different from those in XRD, and small differences in the structures of the Colle Cimino and Pollena samples are possible.

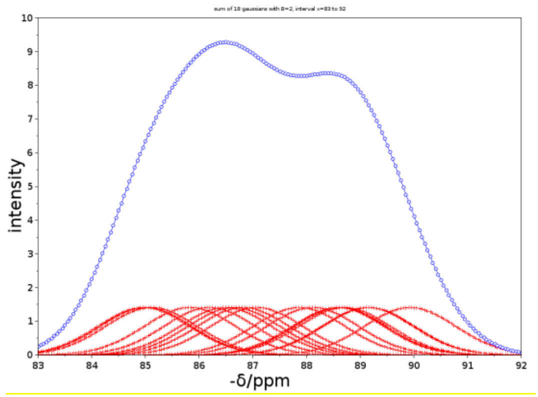

Figure S2.3. The central doublet in the ^{29}Si MAS-NMR spectrum of kaliophilite as calculated *ab initio* from CASTEP. Gaussian curves with $FWHM = 2$ for the 18 single contributions have been used.

References

Clark, S. J., Segall, M. D., Pickard, C. J., Hasnip, P. J., Probert, M. J., Refson, K. & Payne, M. C. (2005). *Z. Kristallogr.* **220**, 567−570.

Gregorkiewitz, M., Li, Y., White, T. J., Withers, R. L. & Sobrados I. (2008). *Can. Mineral.* **46**, 1511−1526.

Sobrados, I. (1991). PhD Thesis, Universidad Complutense, Madrid (in Spanish).

Stebbins, J. F., Murdoch, J. B., Carmichael, I. S. E. & Pines, A. (1986). *Phys. Chem. Mineral.* **13**, 371−381.

S3. Optical microscopy and Weissenberg X-ray diffraction patterns

On the search of possibly pure single crystals, about 12 optically homogeneous microcrystals could be isolated under the petrographic microscope (Fig. S3.1). With an approximate thickness of 40 µm, the observed interference colours indicate $\Delta n \simeq -0.004$, in agreement with literature (Table S1.1). Although clear, many crystals show lines along the needle which are probably twin planes (see also similar observations in Cellai *et al.*, 1992), but artefacts cannot be excluded for such small dimensions.

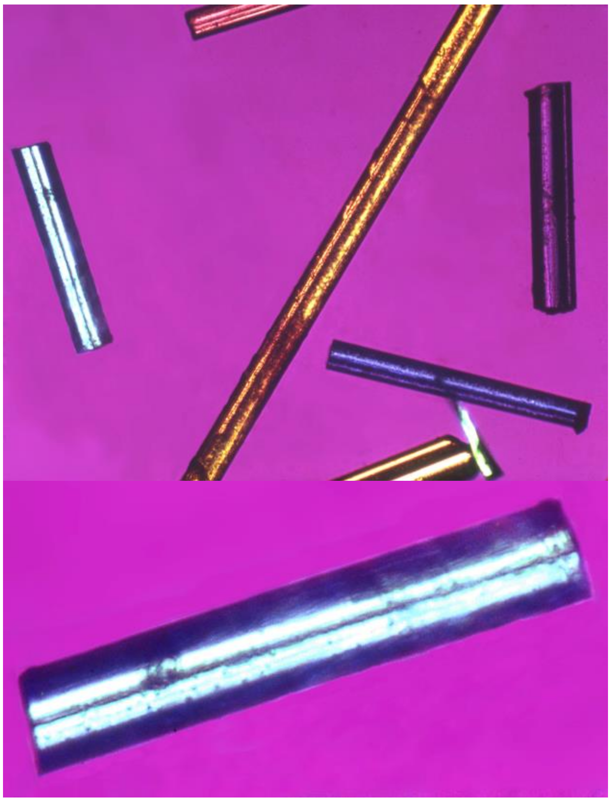

Figure S3.1. Microscopic view of kaliophilite needles from Colle Cimino (crossed polarizers, red I auxiliary plate). The diameter of the magnified needle is about 40 µm. Prismatic faces are clearly visible. In the termination of the hexagonal prisms, basal faces appear to prevail over pyramidal faces.

Further screening of these crystals with laboratory single crystal diffraction revealed that many of them were composed of slightly (~1°) disoriented individuals but no streaks parallel or orthogonal to **c** could be detected. A representative Weissenberg pattern is given in Fig. S3.2.

For one crystal, Cu*K*α Weissenberg photographs of levels *hkl*: $0 \leq l \leq 5$ showed no splitting. In order to test symmetry and twinning, this crystal was used to collect high resolution diffraction scans (Supporting Information S4).

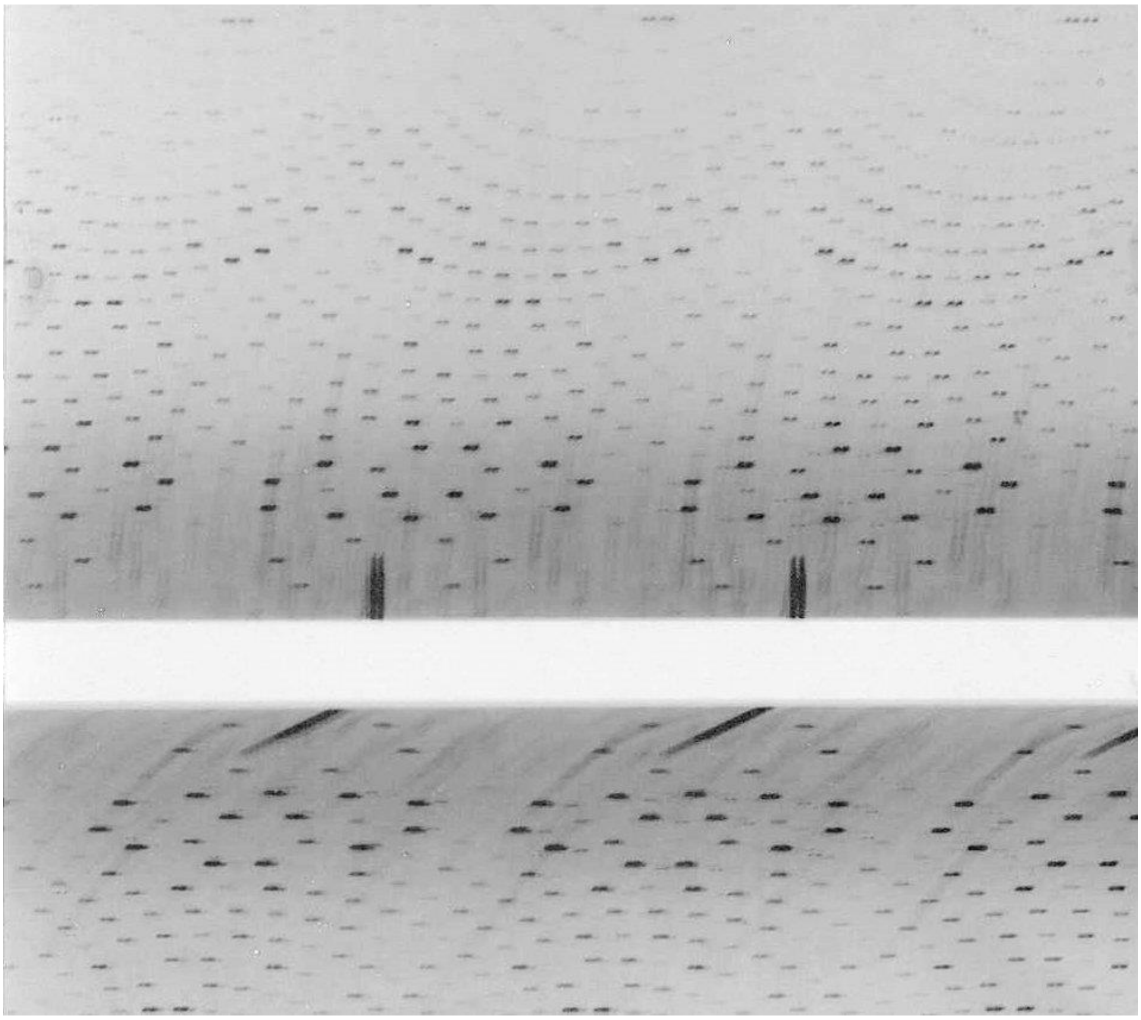

Figure S3.2. Level *hk*1 Weissenberg equi-inclination diagram obtained with Ni-filtered CuK radiation (λ = 1.5406 Å, camera *R* = 27.65 mm, 2º(ω)/mm translation ||**c**) for a kaliophilite 'single' crystal. Abscissa gives crystal rotation, ordinate gives diffraction angle. The **a*** repeat lies on the almost extinct line passing through the abscissa. Splitting of reflections shows that the 'crystal' is composed of essentially two individuals which are rotated by Δω < 1.4º against each other. Note that streaks are due to white radiation and not to structural features.

References

Cellai, D., Carpenter, M. A. & Heaney, P. J. (1992). *Eur. J. Mineral.* **4**, 1209–1220.

S4. High resolution synchrotron single crystal diffraction scans

1D ω scans were performed for 63 complete sets of lattice equivalent reflections. In addition, 3D Q scans were obtained for three sharp reflections (00-10, 4-5-2, 41-2). Fig. S4.1, Fig. S4.2 and Fig. S4.3 show three sets of ω scans, while Fig. S4.4 shows one Q scan.

Confirming preliminary findings (Gregorkiewitz *et al.*, 1991), reflections with parity $h - k = 3n$ appear systematically sharp ($0.008° < FWHM < 0.012°$) with approximately Gaussian shape while the remainder ($h - k \neq 3n$) shows $FWHM \simeq 0.035°$ with a more Lorentzian shape.

With the available data (about 620 reflections in 63 complete sets, about half of which had a signal/background ratio < 3), no obvious violations of Laue symmetry $P6/mmm$ could be observed.

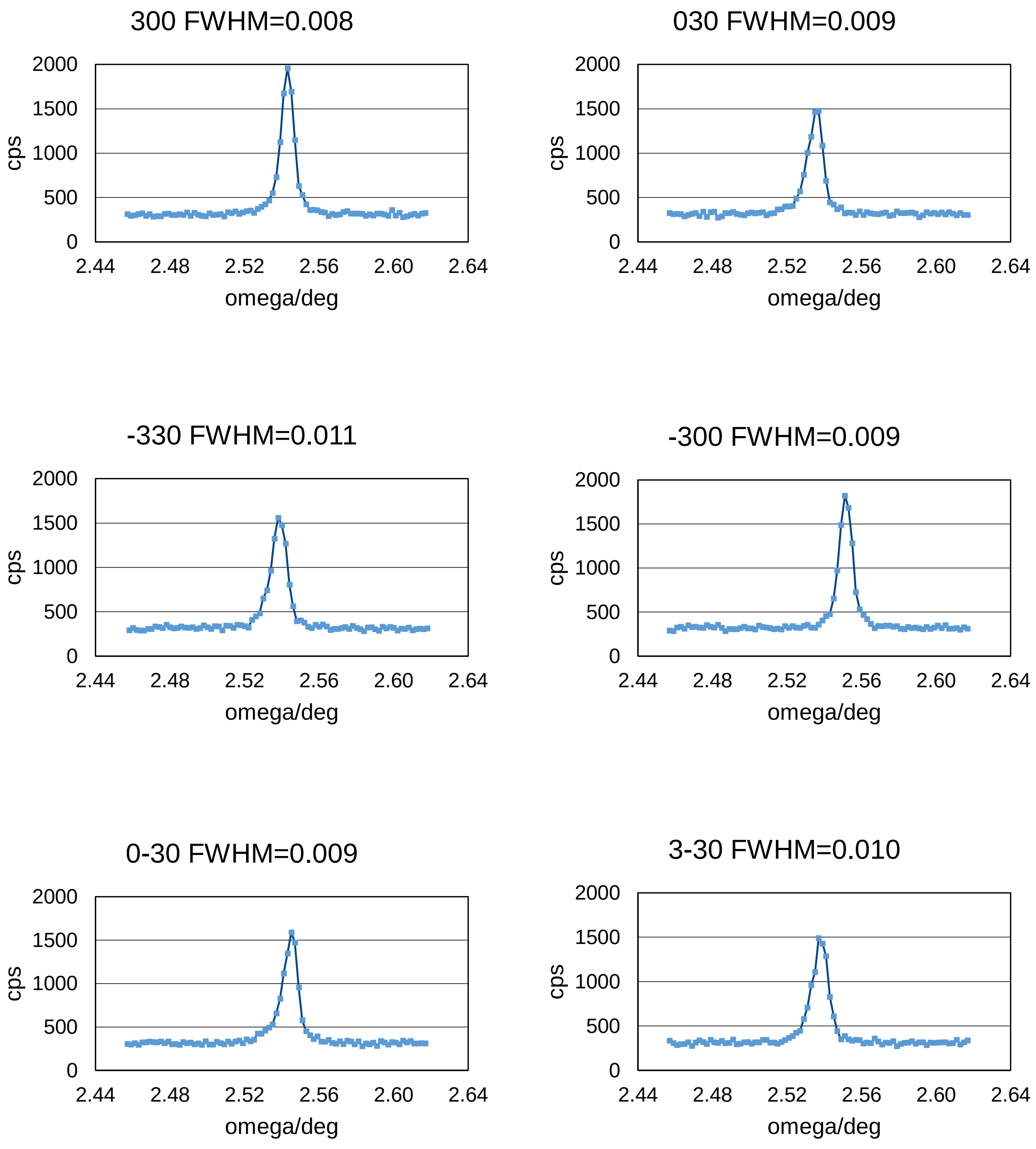

Figure S4.1. Scans obtained in the 6 lattice equivalent positions of reflection 300 ($h - k = 3n$; Friedel equivalents included).

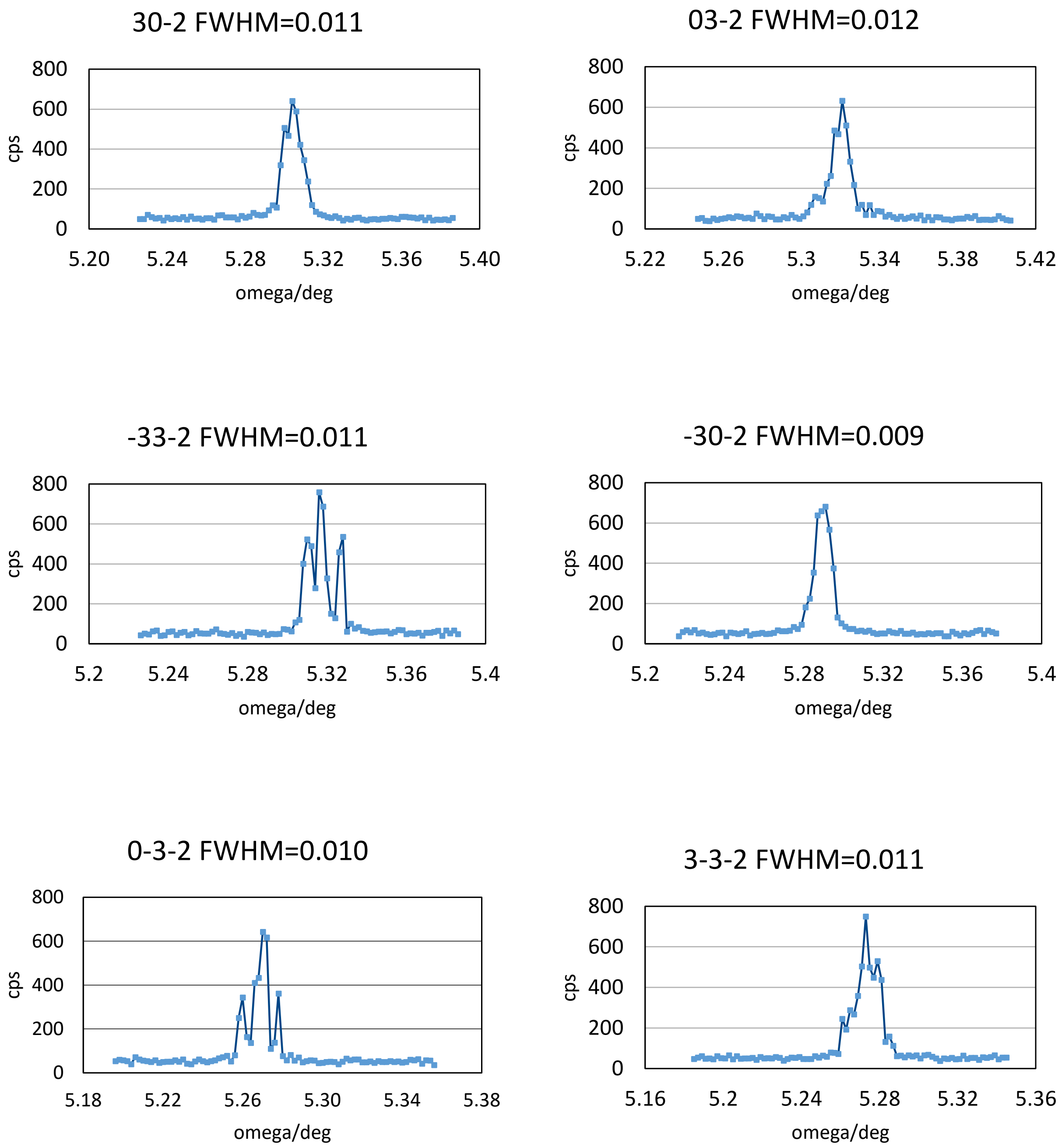

Figure S4.2. Scans obtained in the 6 lattice equivalent positions of reflection 30-2 ($h - k = 3n$; Friedel equivalents ignored).

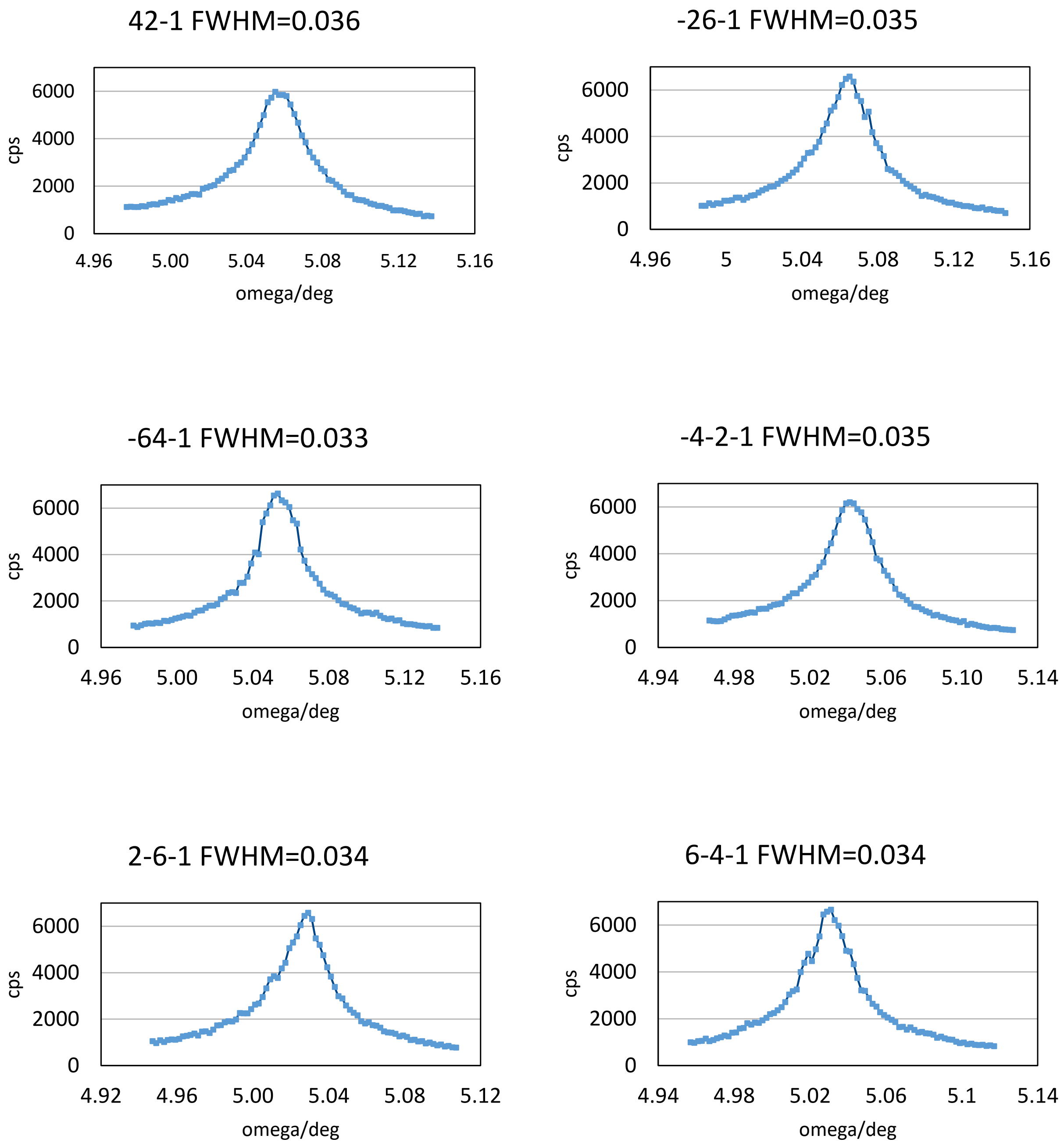

(Figure S4.3 continues in the next page)

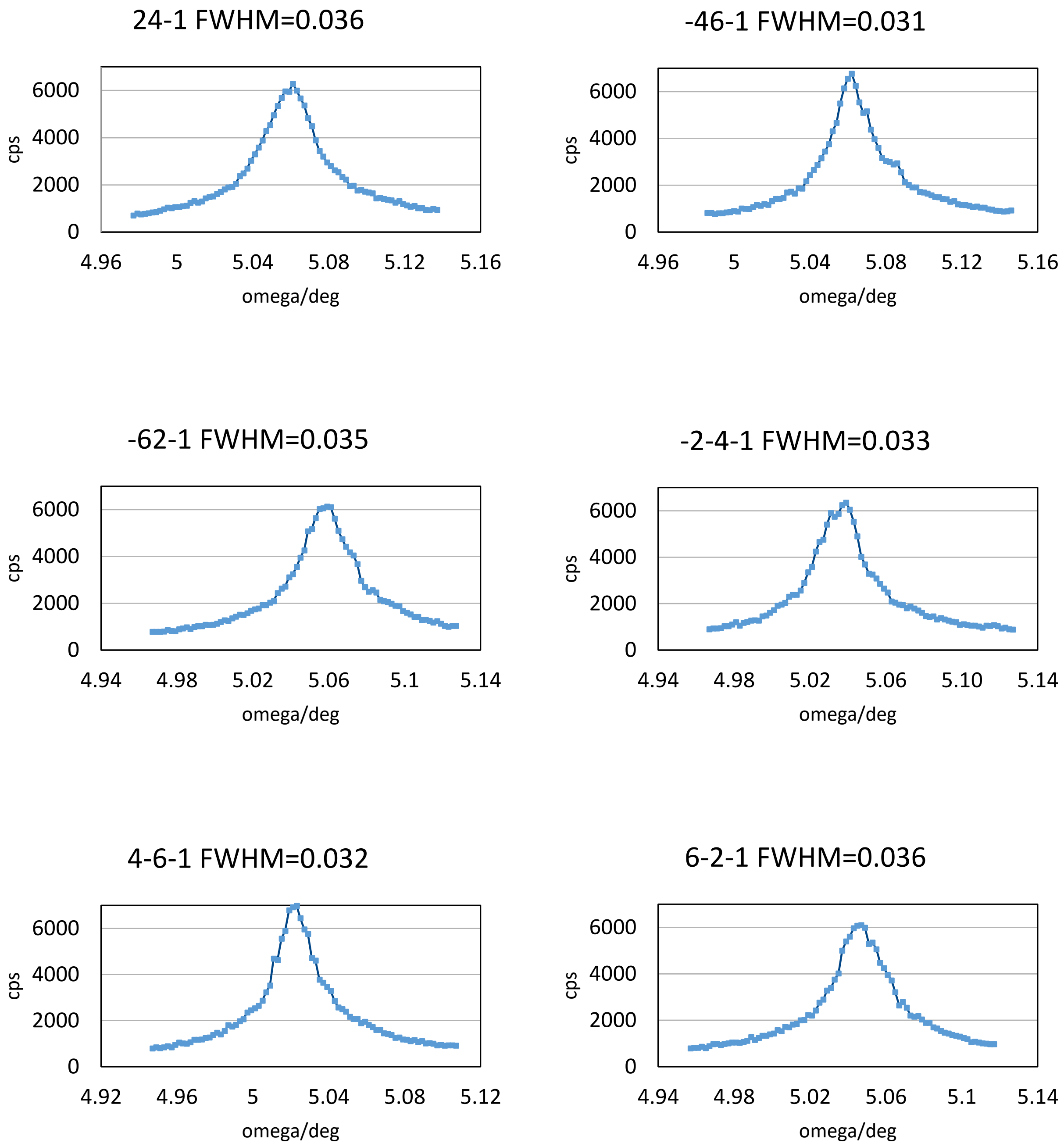

Figure S4.3. Scans obtained in the 12 lattice equivalent positions of reflection 42-1 ($h - k \neq 3n$; Friedel equivalents ignored).

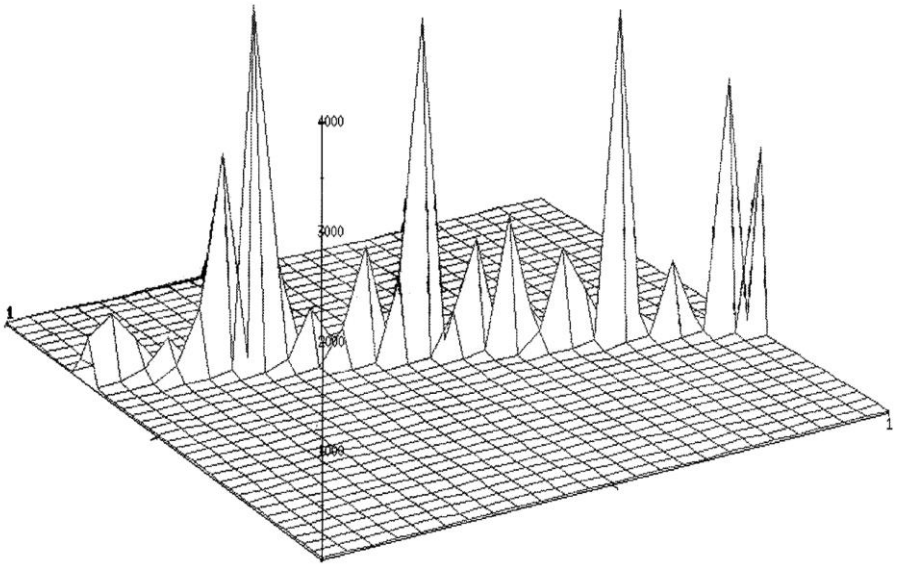

Figure S4.4. 3D representation of one layer of Q scan for reflection 4-5-2 ($h - k = 3n$; step size: $\delta h = \delta k$ ~0.005 Å^{-1}).

References

Gregorkiewitz, M., Artioli, G., Bessière, M. & Elkaim, E. (1991). International Meeting on Synchrotron Radiation in Crystallography, Trieste, Italy, p. 36.

S5. Crystal data, atom parameters, T-O and K-O interatomic distances

Table S5.1. Experimental details and crystallographic data related with the kaliophilite model obtained by 3D ED in space group *P*3*c*1.

**Crystal data**	
Chemical formula	$Al_9K_9Si_9O_{36}$
Crystal system, space group	Trigonal, *P*3*c*1
Temperature (K)	293
a, *c* (Å)	27.1 (5), 8.6 (2)
Z	6
**Data collection**	
Radiation type, λ (Å)	Electrons, 0.0335
Type	Step-wise with precessing beam (1° precession angle)
Tilt range (°), tilt step (°)	115, 1
Ab initio **structure determination**	
No. of measured, independent reflections	11743, 1085
$R_{int}(F)$	0.266
$(\sin \theta/\lambda)_{max}$ (Å^{-1})	1.00
Reflection completeness at 1.00 Å^{-1} (%)	99
Computer programs	*SIR2014* (Burla *et al.*, 2015)
**Structure refinement**	
No. of measured, independent, and observed [$I > 2\sigma(I)$] reflections	11019, 3754, 2764
$R[F^2 > 4\sigma(F^2)]$, $wR(F^2)$, Goof	0.350, 0.641, 2.00
No. of parameters	193
Computer programs	*SHELXL* (Sheldrick, 2015)

Table S5.2. Experimental details and crystallographic data for the single crystal X-ray diffraction refinement of kaliophilite from Pollena (Vesuvius area, Naples, Italy) in space group *P*3.

**Crystal data**	
Chemical formula	$K_{8.22}Na_{0.78}Si_9Al_9O_{36}$
Crystal system, space group	Trigonal, *P*3
Temperature (K)	293
a, *c* (Å)	27.0597 (16), 8.5587 (6)
V ($Å^3$)	5427.3 (7)
Radiation type	Mo*K*α
μ (mm^{-1})	1.68
Crystal size (mm)	0.1 × 0.15 × 0.30
**Data collection**	
Diffractometer	Bruker Smart Breeze diffractometer equipped with an air-cooled CCD detector
Absorption correction	Multi-scan
T_{min}, T_{max}	0.523, 0.747
No. of measured, independent, and observed [$I > 2\sigma(I)$] reflections	127892, 37707, 33165
R_{int}	0.061
$(\sin \theta/\lambda)_{max}$ ($Å^{-1}$)	0.859
**Structure refinement**	
$R[F^2 > 2\sigma(F^2)]$, $wR(F^2)$, S	0.0768, 0.1822, 1.02
No. of reflections	37707
No. of parameters	589
No. of restraints	1
Weight scheme	$w = 1/[\sigma^2(F_o^2) + (0.0519P)^2 + 59.5879P]$ where $P = (F_o^2 + 2F_c^2)/3$
$\Delta\rho_{max}$, $\Delta\rho_{min}$ (e $Å^{-3}$)	2.29, −1.54
Absolute structure	Twinning involves inversion, so Flack parameter cannot be determined
Twin law, twin fraction	(1-10), α = 0.45
Computer programs	SHELXL (Sheldrick, 2015)

Table S5.3. Atomic coordinates (× 10^4) and equivalent isotropic displacement parameters ($Å^2$ × 10^3) for kaliophilite. U_{eq} is used only for K atoms when no K-Na substitution occurs (site 4B) and is defined as one third of the trace of the orthogonalized U_{ij} tensor.

Atom	x	y	z	U_{eq}/U_{iso}	Atom	x	y	z	U_{eq}/U_{iso}
Si1	992(1)	1114(1)	4140(3)	7(1)	Si1B	-115(1)	1079(1)	-857(3)	7(1)
Si2	5574(1)	3332(1)	4128(3)	5(1)	Si2B	5581(1)	2254(1)	-879(3)	4(1)
Si3	3344(1)	5575(1)	5304(3)	8(1)	Si3B	2237(1)	5557(1)	302(3)	9(1)
Si4	2231(1)	2104(1)	-875(3)	7(1)	Si4B	2397(1)	285(1)	4071(3)	7(1)
Si5	3414(1)	2278(1)	4136(3)	6(1)	Si5B	3572(1)	1267(1)	-896(3)	6(1)
Si6	4496(1)	3435(1)	296(3)	8(1)	Si6B	4558(1)	978(1)	5284(3)	9(1)
Si7	4361(1)	4477(1)	5296(3)	8(1)	Si7B	238(1)	4612(1)	305(3)	8(1)
Si8	3153(1)	4329(1)	-803(3)	6(1)	Si8B	1194(1)	4285(1)	4187(3)	5(1)
Si9	2110(1)	3130(1)	4173(3)	8(1)	Si9B	954(1)	3044(1)	-880(3)	7(1)
Al1	992(1)	1115(1)	345(3)	4(1)	Al1B	-116(1)	1081(1)	5357(3)	5(1)
Al2	5572(1)	3334(1)	351(3)	3(1)	Al2B	5580(1)	2254(1)	5352(3)	2(1)
Al3	3343(1)	5573(1)	-905(3)	6(1)	Al3B	2232(1)	5557(1)	4098(3)	7(1)
Al4	2228(1)	2100(1)	5343(3)	3(1)	Al4B	2398(1)	288(1)	399(3)	3(1)
Al5	3414(1)	2278(1)	341(3)	4(1)	Al5B	3576(1)	1273(1)	5360(3)	3(1)
Al6	4495(1)	3436(1)	4119(3)	6(1)	Al6B	4562(1)	978(1)	-860(3)	6(1)
Al7	4359(1)	4476(1)	-890(3)	7(1)	Al7B	242(1)	4619(1)	4112(3)	6(1)
Al8	3147(1)	4325(1)	5315(3)	4(1)	Al8B	1197(1)	4284(1)	318(3)	3(1)
Al9	2102(1)	3122(1)	321(3)	6(1)	Al9B	951(1)	3040(1)	5374(3)	3(1)
Na1	0	0	2281(8)	6(1)	K1B	0	0	7245(6)	18(1)
K2	6667	3333	2233(6)	20(1)	K2B	6667	3333	7230(5)	13(1)
K3	3333	6667	2203(5)	12(1)	K3B	3333	6667	7171(7)	26(1)
K4	2197(1)	1255(1)	2240(3)	14(1)	K4B	2405(1)	1107(1)	7240(3)	15(1)
					Na4B	2255(10)	1209(9)	7240(30)	15(1)
K5	4521(1)	1944(1)	2227(3)	16(1)	K5B	4537(1)	2571(1)	7220(3)	18(1)
K6	5586(1)	4546(1)	2217(3)	12(1)	K6B	5741(1)	1140(1)	7209(3)	19(1)

K7	4090(1)	5296(1)	2223(3)	16(1)	K7B	1205(1)	5258(1)	7228(3)	13(1)
K8	2375(1)	4455(1)	2241(3)	17(1)	K8B	2080(1)	4356(1)	7238(3)	17(1)
K9	855(1)	2138(1)	2243(3)	16(1)	Na9B	1125(2)	2069(2)	7256(5)	8(1)
K10	3326(1)	3408(1)	2222(3)	19(1)	K10B	108(2)	3429(2)	7230(4)	36(1)
K11	3310(1)	3210(1)	7230(3)	13(1)	K11B	-152(1)	3308(1)	2221(4)	24(1)
O1	821(4)	1046(4)	2256(12)	24(2)	O1B	-218(5)	1133(5)	7269(13)	29(2)
O2	5691(3)	3533(3)	2247(7)	6(1)	O2B	5487(3)	2360(3)	7228(8)	8(1)
O3	3127(4)	5490(4)	7197(11)	23(2)	O3B	2111(4)	5638(4)	2201(12)	22(2)
O4	2040(3)	2010(3)	7251(9)	14(1)	O4B	2626(3)	504(3)	2247(8)	10(1)
O5	3264(3)	2314(3)	2244(9)	13(1)	O5B	3737(3)	1468(3)	7230(8)	11(1)
O6	4481(3)	3312(4)	2197(10)	17(1)	O6B	4492(4)	815(4)	7160(10)	19(2)
O7	4335(4)	4320(4)	7200(11)	19(2)	O7B	387(3)	4785(3)	2200(9)	14(1)
O8	3121(3)	4296(3)	7272(9)	14(1)	O8B	1165(3)	4164(3)	2267(8)	9(1)
O9	2014(4)	3044(4)	2259(11)	23(2)	O9B	758(3)	2862(3)	7271(10)	15(1)
O10	614(3)	1333(3)	5126(9)	15(1)	O10B	581(3)	1327(3)	-655(8)	11(1)
O11	864(4)	494(4)	4718(11)	19(2)	O11B	876(3)	456(4)	-301(10)	15(1)
O12	3713(3)	1878(3)	4334(8)	10(1)	O12B	3710(3)	1846(3)	123(9)	13(1)
O13	5381(3)	2649(3)	4238(8)	13(1)	O13B	5384(3)	2625(3)	211(8)	10(1)
O14	6179(3)	3718(3)	5114(8)	11(1)	O14B	6248(3)	2455(3)	-632(8)	9(1)
O15	4769(3)	5177(3)	5075(8)	10(1)	O15B	410(3)	5211(3)	-626(9)	15(1)
O16	4013(4)	6054(4)	5118(10)	18(2)	O16B	2047(4)	5958(4)	-708(10)	17(1)
O17	2945(4)	5776(4)	4362(11)	22(2)	O17B	2900(3)	5769(3)	50(9)	14(1)
O18	1467(4)	2871(3)	4903(9)	16(1)	O18B	1435(3)	2858(3)	-438(9)	14(1)
O19	1685(4)	1632(4)	206(10)	16(1)	O19B	1815(4)	322(4)	4358(10)	20(2)
O20	1667(3)	1606(3)	4300(9)	13(1)	O20B	1780(4)	314(4)	134(10)	18(1)
O21	5043(3)	3418(3)	-490(8)	12(1)	O21B	5168(3)	1540(3)	4854(9)	15(1)
O22	5062(3)	3423(3)	4943(8)	11(1)	O22B	5197(3)	1571(3)	-398(9)	14(1)
O23	3224(3)	4943(3)	-130(8)	8(1)	O23B	1801(3)	4861(3)	4697(8)	10(1)

O24	3224(3)	4960(3)	4603(8)	9(1)	O24B	1833(3)	4892(3)	-233(8)	12(1)
O25	2772(3)	1992(3)	-668(9)	13(1)	O25B	2890(4)	742(3)	5290(10)	17(1)
O26	2800(3)	1996(3)	5132(8)	9(1)	O26B	2911(4)	762(4)	-826(10)	17(1)
O27	3875(3)	2968(3)	-433(9)	13(1)	O27B	4012(3)	1032(3)	4623(9)	15(1)
O28	3860(3)	2924(3)	4856(8)	9(1)	O28B	3988(3)	1031(3)	-167(9)	14(1)
O29	4629(4)	4102(4)	15(10)	17(1)	O29B	4554(3)	459(3)	4272(8)	13(1)
O30	4627(3)	4126(3)	4391(8)	10(1)	O30B	4554(3)	431(3)	159(9)	14(1)
O31	3667(3)	4237(3)	-173(9)	12(1)	O31B	655(3)	4350(3)	4825(9)	13(1)
O32	3711(3)	4261(3)	4581(7)	6(1)	O32B	630(3)	4349(3)	-368(8)	9(1)
O33	2542(3)	3841(3)	-117(9)	15(1)	O33B	1193(3)	3764(3)	5168(8)	13(1)
O34	2514(3)	3808(3)	4549(8)	10(1)	O34B	1193(3)	3737(3)	-680(7)	7(1)
O35	2444(3)	2767(3)	-331(9)	15(1)	O35B	370(4)	2644(4)	4230(10)	18(1)
O36	2427(3)	2795(3)	4848(9)	14(1)	O36B	400(4)	2687(4)	221(9)	16(1)

Table S5.4. Bond lengths (Å) for the framework of kaliophilite.

Si1	O11	1.611(9)	Si2	O13	1.654(8)	Si3	O16	1.623(9)
	O20	1.641(8)		O14	1.666(8)		O24	1.640(7)
	O10	1.650(8)		O2	1.674(7)		O17	1.643(10)
	O1	1.663(10)		O22	1.678(8)		O3	1.700(10)
	average	1.641(22)		average	1.668(11)		average	1.652(34)
Si4	O25	1.646(8)	Si5	O12	1.651(8)	Si6	O27	1.639(8)
	O35	1.654(9)		O28	1.668(7)		O21	1.647(8)
	O4#5	1.665(9)		O26	1.673(7)		O6	1.658(9)
	O19	1.668(9)		O5	1.685(8)		O29	1.673(9)
	average	1.658(10)		average	1.669(14)		average	1.654(15)
Si7	O30	1.643(7)	Si8	O31	1.623(8)	Si9	O34	1.630(8)
	O15	1.660(8)		O33	1.624(8)		O36	1.632(8)
	O32	1.667(7)		O8#5	1.649(8)		O18	1.639(9)
	O7	1.676(9)		O23	1.678(7)		O9	1.657(10)
	average	1.662(14)		average	1.644(26)		average	1.640(12)
Si1B	O11B#1	1.615(9)	Si2B	O14B	1.617(7)	Si3B	O17B	1.602(9)
	O20B#1	1.635(9)		O13B	1.643(7)		O24B	1.636(8)
	O1B#5	1.647(11)		O22B	1.655(8)		O16B	1.656(9)
	O10B	1.662(8)		O2B#5	1.687(7)		O3B	1.697(10)
	average	1.640(20)		average	1.651(29)		average	1.648(40)
Si4B	O35B#9	1.636(9)	Si5B	O26B	1.620(9)	Si6B	O21B	1.632(9)
	O19B	1.647(9)		O28B	1.667(8)		O29B	1.646(8)
	O25B	1.657(9)		O12B	1.666(8)		O6B	1.651(9)
	O4B	1.675(7)		O5B#5	1.680(7)		O27B	1.655(8)
	average	1.654(17)		average	1.658(26)		average	1.646(10)
Si7B	O30B#1	1.628(8)	Si8B	O33B	1.638(8)	Si9B	O36B	1.618(9)
	O32B	1.647(7)		O31B	1.649(8)		O18B	1.657(9)

	O15B	1.652(8)		O23B	1.661(7)		O34B	1.658(7)
	O7B	1.680(8)		O8B	1.669(7)		O9B#5	1.664(8)
	average	1.652(21)		average	1.654(14)		average	1.649(21)
Al1	O1	1.685(10)	Al2	O2	1.690(7)	Al3	O23	1.703(7)
	O19	1.692(9)		O21	1.717(8)		O3#5	1.703(10)
	O10B	1.713(8)		O13B	1.725(7)		O16B#3	1.709(9)
	O11B	1.740(9)		O14B#2	1.731(7)		O17B	1.738(9)
	average	1.708(25)		average	1.716(18)		average	1.713(17)
Al4	O20	1.692(8)	Al5	O5	1.693(8)	Al6	O6	1.675(9)
	O4	1.691(9)		O12B	1.725(8)		O28	1.700(7)
	O26	1.717(7)		O25	1.735(8)		O22	1.704(8)
	O36	1.730(8)		O27	1.775(8)		O30	1.734(8)
	average	1.708(19)		average	1.732(34)		average	1.703(24)
Al7	O7#5	1.681(9)	Al8	O8	1.677(8)	Al9	O9	1.674(10)
	O29	1.703(9)		O34	1.709(8)		O18B	1.703(8)
	O15B#3	1.720(9)		O24	1.737(7)		O35	1.726(8)
	O31	1.756(8)		O32	1.738(7)		O33	1.741(9)
	average	1.715(32)		average	1.715(29)		average	1.711(29)
Al1B	O1B	1.677(11)	Al2B	O2B	1.671(7)	Al3B	O3B	1.692(10)
	O19B#1	1.705(9)		O21B	1.734(8)		O23B	1.725(8)
	O10	1.750(8)		O14	1.704(8)		O16	1.725(10)
	O11	1.753(9)		O13	1.706(8)		O17	1.716(10)
	average	1.721(37)		average	1.704(26)		average	1.715(16)
Al4B	O4B	1.692(7)	Al5B	O5B	1.674(7)	Al6B	O30B	1.708(8)
	O26B	1.701(9)		O25B	1.687(9)		O22B	1.711(8)
	O20B	1.724(9)		O27B	1.730(8)		O28B	1.735(8)
	O36B#9	1.736(9)		O12	1.727(7)		O6B#5	1.737(9)
	average	1.713(20)		average	1.704(28)		average	1.722(15)

Al7B	O7B	1.690(8)	Al8B	O8B	1.694(7)	Al9B	O35B	1.702(9)
	O29B#1	1.713(8)		O34B	1.704(7)		O9B	1.700(9)
	O31B	1.722(8)		O32B	1.733(8)		O33B	1.739(8)
	O15	1.696(78)		O24B	1.748(8)		O18	1.720(9)
	average	1.705(15)		average	1.720(25)		average	1.715(18)

#1 -y,x-y,z; #2 -x+y+1,-x+1,z; #3 -y+1,x-y+1,z; #4 -y+1,x-y+1,z+1; #5 x,y,z-1; #6 -x+y+1,-x+1,z-1; #7 -y+1,x-y+1,z-1; #8 x,y,z+1; #9 -x+y,-x,z; #10 -y+1,x-y,z; #11 -x+y,-x+1,z; #12 -y,x-y,z-1; #13 -x+y,-x,z-1; #14 -y,x-y,z+1; #15 -x+y,-x,z+1; #16 -y+1,x-y,z+1; #17 -x+y+1,-x+1,z+1; #18 -x+y,-x+1,z+1; #19 -y+1,x-y,z-1 #20 -x+y,-x+1,z-1

Table S5.5 Bond lengths (Å) for the extra-framework cations in kaliophilite.

Na1	O1	2.580(10) x 3	K1B	O11B	2.938(9) X 3			
	O11	2.911(10) X 3		O1B	3.398(11) X 3			
K2	O2	2.949(6) X 3	K2B	O14	2.734(8) X 3			
	O14B	3.201(8) X 3		O14B	2.754(7) X 3			
	O14	3.205(8) X 3		O2B	2.953(6) X 3			
K3	O17B	2.797(8) X 3	K3B	O3	2.944(10) X 3			
	O17	2.792(10) X 3		O17	3.188(10) X 3			
	O3B	3.079(9) X 3		O17B	3.240(9) X 3			
K4	O19	2.720(9)	K4B	O26B	2.601(9)	Na4B	O4	2.51(2)
	O20	2.727(8)		O25B	2.604(9)		O1B	2.57(2)
	O4B	2.799(7)		O1B	2.658(11)		O25	2.59(2)
	O19B	2.850(9)		O25	2.747(8)		O26	2.61(2)
	O20B	2.851(9)		O26	2.759(8)		O25B	3.08(3)
	O5	2.877(8)		O4	3.058(9)		O26B	3.08(3)
	O25	3.081(8)		O19B	3.124(9)			
	O26	3.089(7)		O20B	3.158(9)			
				O5B	3.231(8)			
K5	O13B	2.744(7)	K5B	O5B	2.671(7)			
	O13	2.753(8)		O21	2.798(8)			
	O12B	2.747(8)		O22	2.803(8)			
	O12	2.770(7)		O2B	2.898(7)			
	O27B	2.966(8)		O28	3.183(7)			
	O28B	2.970(8)		O27	3.213(8)			
	O21B	3.352(8)		O12	3.227(7)			
	O22B	3.355(8)		O12B	3.262(8)			
				O13	3.361(8)			
				O13B	3.390(7)			

K6	O29B	2.770(8)	K6B	O22	2.765(7)
	O30B	2.782(8)		O21	2.791(8)
	O2	2.892(6)		O7	2.965(9)
	O22B	2.926(8)		O6B	3.039(9)
	O21B	2.927(8)		O21B	3.057(9)
	O30	2.923(7)		O29	3.067(8)
	O29	2.931(9)		O22B	3.074(8)
	O6	3.180(9)		O30	3.092(7)
K7	O7B	2.727(8)	K7B	O16B	2.754(9)
	O23	2.866(7)		O15B	2.783(8)
	O24	2.885(7)		O16	2.794(9)
	O3B	2.887(10)		O15	2.810(7)
	O15	3.168(7)		O31B	2.970(8)
	O15B	3.177(8)		O32B	2.981(7)
	O32	3.179(7)		O23B	3.191(7)
	O31	3.233(8)		O24B	3.205(8)
	O16	3.288(9)			
	O16B	3.346(9)			
K8	O34	2.787(8)	K8B	O33B	2.760(8)
	O33	2.790(8)		O34B	2.777(7)
	O23	2.848(7)		O23B	2.863(8)
	O24	2.845(7)		O24B	2.865(8)
	O8B	2.959(7)		O8	2.901(8)
	O23B	3.120(7)		O3	2.958(10)
	O24B	3.125(8)		O33	3.219(8)
				O34	3.259(8)
K9	O9	2.856(10)	Na9B	O10	2.538(9)
	O35B	2.880(9)		O10B	2.537(8)

	O1	2.909(10)		O4	2.559(9)
	O18B	2.910(8)		O18B	2.716(8)
	O18	2.928(8)		O18	2.760(9)
	O36B	2.924(9)		O9B	2.779(9)
	O10	3.135(8)			
	O10B	3.145(8)			
	O20B	3.289(9)			
	O19B	3.302(9)			
K10	O31	2.833(8)	K10B	O9B	2.854(8)
	O32	2.843(7)		O6B	2.893(9)
	O5	2.877(8)		O32B	2.983(8)
	O35	3.056(9)		O31B	2.992(9)
	O36	3.112(8)		O33B	3.146(8)
	O9	3.177(10)		O34B	3.173(7)
	O27	3.248(8)			
	O6	3.261(8)			
	O28	3.280(7)			
K11	O27	2.788(8)	K11B	O4B	2.855(7)
	O28	2.844(7)		O28B	2.912(9)
	O7	2.897(9)		O27B	2.928(9)
	O36	2.906(8)		O8B	3.133(8)
	O35	2.910(8)		O29B	3.174(8)
	O8	3.226(8)		O30B	3.196(8)
	O31	3.304(8)		O36B	3.240(9)
	O4	3.345(8)		O35B	3.273(9)
	O32	3.365(7)		O32B	3.369(8)
	O26	3.374(7)		O31B	3.396(8)

References

Burla, M. C., Caliandro, R., Carrozzini, B., Cascarano, G.L., Cuocci, C., Giacovazzo, C., Mallamo, M., Mazzone, A. & Polidori G. (2015). *J. Appl. Cryst.* **48**, 306−309.

Sheldrick, G. M. (2015). *Acta Cryst.* C**71**, 3–8.

S6. Rietveld refinements using high resolution synchrotron radiation

The major steps of Rietveld refinements are resumed in Table S6.1 and can be divided in two groups: the first, from #40 to #235, starts with the raw model in space group *P*3*c*1 obtained from 3D ED and addresses various improvements until the introduction of the model in *P*3 obtained from single crystal refinement, and the second, from #b10 to #e20, is concerned with the correct description of peak shape (anisotropic line broadening ALB). Some representative patterns are shown at the end.

Table S6.1. Summary of Rietveld and Le Bail refinements of kaliophilite using high resolution synchrotron powder diffraction data. Peak shape is TCH pseudoVoigt throughout.

#	prog	sg	Ks	*NP*	parameters (refined/not refined)	χ^2	$R(F^2)$	conv	parity for *h* - *k*
40	gsas	*P*3*c*1	-	33	*a-Z-X-Y* 24*bk* 3*U*	935	0.34	Y	
58	"	"	-	195	*a-Z-X-Y* 24*bk* 3*U*-162*x*(DLS)	444	0.20	N	
642	"	"	3.1	127	*a-Z-X-Y-st-pt-sf* 32*bk* 3*U*-80*x*(DLS)	357	0.17	N	
235	"	*P*3	2.7	44	*a-Z-X-Y-st-pt-sf* 32*bk*	292	0.131	Y	
b10	FP	"	2.3	38	*a-Z-X-Y-*Y_{anis} 27*bk*	136	0.090	Y	*B*: 3*n*
c18	"	"	3.0	10 LB	*a-Z-X-Y-*Y_{anis}-ʃ 51*bk*	43.1	0.00018	Y	*B*-ʃ: 3*n*
f21	"	"	2.8	13 LB	*a-Z-X-Y-*Y_{anis}-ʃ 45*bk*	31.3	0.00034	Y	*B*: 3*n*[+1], ʃ: 3*n*
e20	"	"	2.8	2	*a-Z-X-Y-*Y_{anis}-ʃ 51*bk* fix from #c18	117	0.089	Y	*B*-ʃ: 3*n*

NOTES: prog program, sg space group, Ks % kalsilite impurity, *NP* number of refined parameters, conv convergence, gsas GSAS (Larson & Von Dreele, 2004), FP FullProf (Rodríguez-Carvajal, 2001), LB Le Bail refinement (rest is Rietveld), *a* unit cell(s), *Z* 2θ-origin, *X*(*Y*) strain(size) component of Lorentzian, *st-pt-sf* shape anisotropy with subcell (gsas), Y_{anis} subcell size component (FP), 24*bk* 24 background parameters, *U* atom displacement parameter, *x* atom coordinates, DLS restraint geometry for tetrahedra, *B FWHM*, ʃ peak shift (*B* and ʃ are the parameters to which the parity condition applies).

*Group 1 (structure from P*3*c*1 to *P*3*)*

The starting structure, obtained from 3D ED and preliminarily refined using electron diffraction intensities, had already an ordered Al/Si distribution. Once introduced in Rietveld refinement (#40, $R(F^2)$ = 0.34), it became immediately clear that there were enormous problems with peak shape, and trials to refine the structure (#58, $R(F^2)$ = 0.20) showed correlations and divergence even when the tetrahedral distances (T-O and O-O) were restrained. Slight improvements could be achieved introducing shape anisotropy to model ALB and a small fraction of kalsilite as impurity to account for some extra peaks (#642, $R(F^2)$ = 0.17), but results correspond to false minima recognized by lack of convergence and improbable interatomic distances.

In the meanwhile, SCXRD refinement using data from a twinned crystal allowed to obtain an improved structure in the subgroup *P*3. These atom parameters, introduced in Rietveld refinement without releasing them, clearly lowered the residual (#235, χ^2 = 292, $R(F^2)$ = 0.131) but the inadequateness of peak shape description became now even more visible and is reflected by the high reduced χ^2 value[a]. The use of a microstrain model (Stephens, 1999) as an alternative to explain ALB was not successful either.

Group 2 (improved modelling of peak shape and final refinement)

Instead, a substantial improvement was obtained (#b10, χ^2 = 136, $R(F^2)$ = 0.090), again without changing atom parameters, if the parity rule *hkl*: $h - k = 3n$ (*hkl*: $h - k \neq 3n$) for sharp (broad) reflections, found from synchrotron single crystal experiments, was introduced.

The new peak shape model was improved in two (structureless) Le Bail refinements (#c18, χ^2 = 43.1; #f21, χ^2 = 31.3), whose details will be discussed below. The overall parameters obtained in #c18 were, unchanged, fed into a final Rietveld refinement (#e20, χ^2 = 117, $R(F^2)$ = 0.089) where only the scale factors of kaliophilite and

[a] Values for χ^2 are high throughout due to the low statistical error of diffracted intensities, a fact well known for work with synchrotron radiation, but χ^2 = 292 is still 10 times higher than the minimum obtained in standard refinement (χ^2 = 30).

kalsilite were allowed to refine. As expected, the global fit error χ^2, but not the structural error $R(F^2)$, dropped with respect to step #b10.

The structural residuals of the final result (#e20, $R(F^2)$ = 0.089, $R(F)$ = 0.071) compare well with their counterparts from single crystal refinement (w$R2$ = 0.182, $R1$ = 0.090), a confirmation which is independent from any bias introduced by twin laws. In addition, Rietveld refinement gave at least three new informations: (a) more representative (whole phase) and precise (synchrotron radiation) values for the unit cell parameters, (b) the detection of kalsilite as an impurity in Colle Cimino kaliophilite, and (c) a wealth of details on microstructure which is discussed below.

Interpretation of line shape and displacement (microstructure)

The description of peak shape and position was addressed, without any bias coming from crystal structure, using full pattern Le Bail refinements (#c18 and #f21 in Table S6.1) as well as single peak fittings of some selected reflections.

In experiment #c18 (χ^2 = 43.1), ALB was simulated using the $h - k = 3n$ parity rule that divides reflections into two groups with different Lorentzian contributions to the line *FWHM*. Inspection of the patterns (e.g. peak 311 in Fig. S6.3) shows that many broad peaks exhibit a super-Lorentzian shape which cannot be simulated in the TCH approach of FullProf. A trial to overcome this limitation was made in experiment #f21 (χ^2 = 31.3) where three groups of reflections ($h - k = 3n$, $h - k = 3n + 1$ and $h - k = 3n - 1$) were defined. With $3n - 1$ and $3n + 1$ being lattice equivalent pairs, this is a mathematical trick to create super-Lorentzians which allow an improved Le Bail fit, showing that super-Lorentzian peak shape explains most of the gap from χ^2 = 43.1 to the minimum at about χ^2 = 30, roughly expected also from NAC standard refinement.

A direct check of the parity rule hkl: $h - k = 3n$ (hkl: $h - k \neq 3n$) for sharp (broad) reflections is shown in Fig. S6.1 which compares calculated and observed total *FWHM*s. The observed line widths (blue dots) were obtained from well resolved reflections and clearly distribute over two branches, confirming that the parity rule holds for all reflections. The lower branch ranges from *FWHM* = 0.013° to 0.034° and closely follows the calculated mean (red diamonds) at the same angle, whereas the upper one (about 0.028 – 0.042°) is more dispersed and values are considerably lower than their calculated counterparts, due to the super-Lorentzian form which, at constant area, has a broader base and smaller *FWHM*.

Outliers can in most cases be explained. As an example, in the upper branch, negative deviations from the mean are seen for various reflections with high --l indices, *e.g.* 213, 423, 515, 875. This indicates needle shaped domains for the 27 Å cell, elongated parallel to **c** in perfect agreement with HRTEM where faults parallel to **c** are observed[b]. A resulting scheme for ALB is given in the drawing at right of Fig. S6.2. Note the difference to the scheme at left which was unable to fit data (experiment #235).

---

[b] The shape anisotropy could be modelled, but was far less important than the parity rule for peak width and the two models cannot be applied simultaneously.

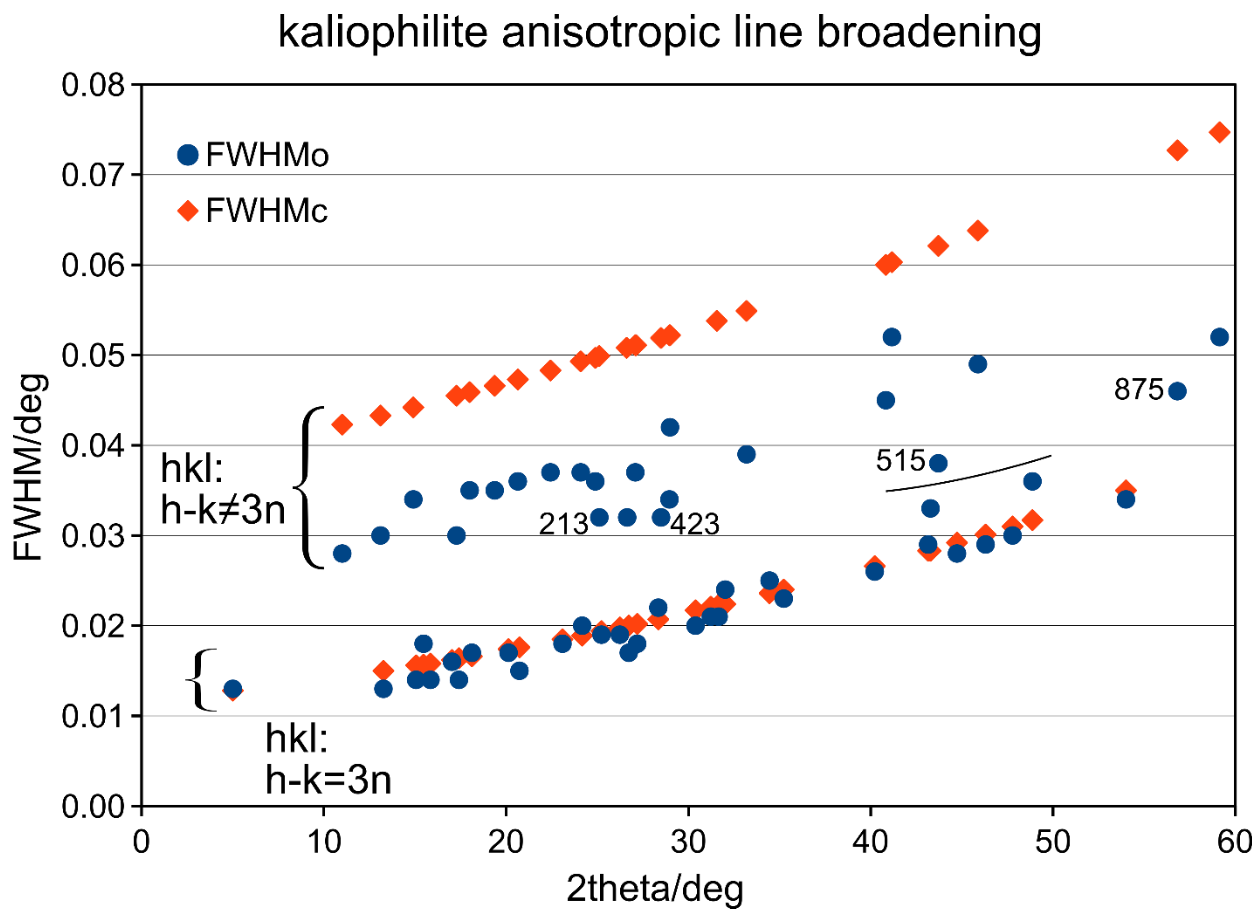

Figure S6.1. Observed (blue) and calculated (#c18; red diamonds) peak widths for kaliophilite high resolution powder diffraction pattern obtained with synchrotron radiation. For both series, reflections complying with *hkl*: $h - k = 3n$ are sharper than the remainder. See text for explanations.

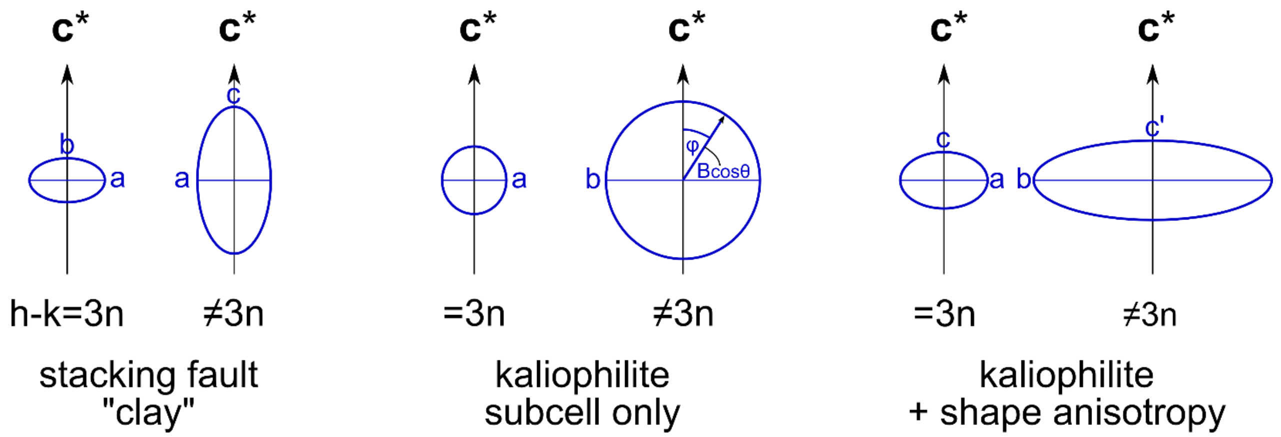

Figure S6.2. Rotational ellipsoids representing anisotropic line broadening (ALB) for three different hypotheses containing a subcell defined by parity condition $h - k = 3n$. The vector $B\cos\theta = FWHM\cos\theta$ is the $\cos\theta$ corrected line breadth for reflections at inclination φ from axis **c***. In the first case (“clay”), there are stacking faults (planes orthogonal to **c***) and the two sets of reflections have the same width orthogonal to **c*** but different widths for $\varphi < 90°$. In the second case (corresponding to the model used in all refinements from #b10 onwards), widths are independent and different but isotropic within each set. The last case represents the most probable model for kaliophilite, where shape anisotropy (needles parallel to **c**) is added. Note that, here, the length of the coherent domain in subcell and full cell are likely to be similar (semiaxes $c \simeq c'$), in contrast to case 1 where the width of reflections perpendicular **c*** is common to the two sets.

The vertical shift (~0.017°) between the two branches is constant, *i.e.* there is a difference in the size component of line broadening while microstrain remains similar. Domain sizes were estimated using the Scherrer equation with integral breadths β obtained from both Le Bail refinement and single peak fits. For the broad reflections, after subtraction of the instrument and Lorentzian strain contributions, one obtains $\beta = 0.050°$ and $\beta = 0.045°$, respectively, corresponding to apparent domain sizes of 1360 Å and 1500 Å. Such domain sizes apply essentially to *hkl*: $h - k \neq 3n$ reflections with low indices (*e.g.* 211 and 311) and an inclination of $\varphi \simeq 50°$ from **c***. Remembering the approximately cylindrical shape anisotropy discussed above (Fig. S6.2), one obtains an average of 1070 Å for the thickness of the cylinders, in fair agreement with HRTEM results where the 27 Å cell is seen to extend over 200-1200 Å in **ab**.

Fig. S6.3 shows the intensity patterns of Le Bail and single peak fits for the representative reflections 311 (*hkl*: $h - k \neq 3n$) and 410 (*hkl*: $h - k = 3n$). Fig. S6.4 reports the best Rietveld fit (#e20, $\chi^2 = 117$).

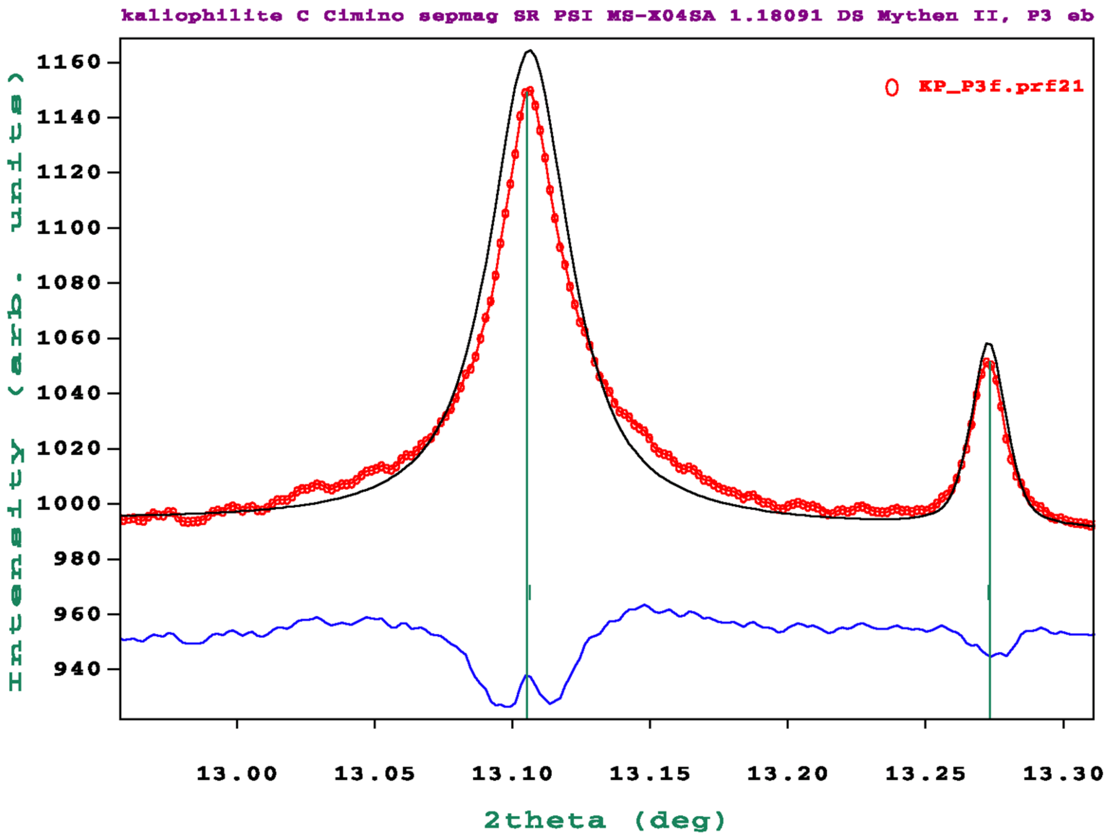

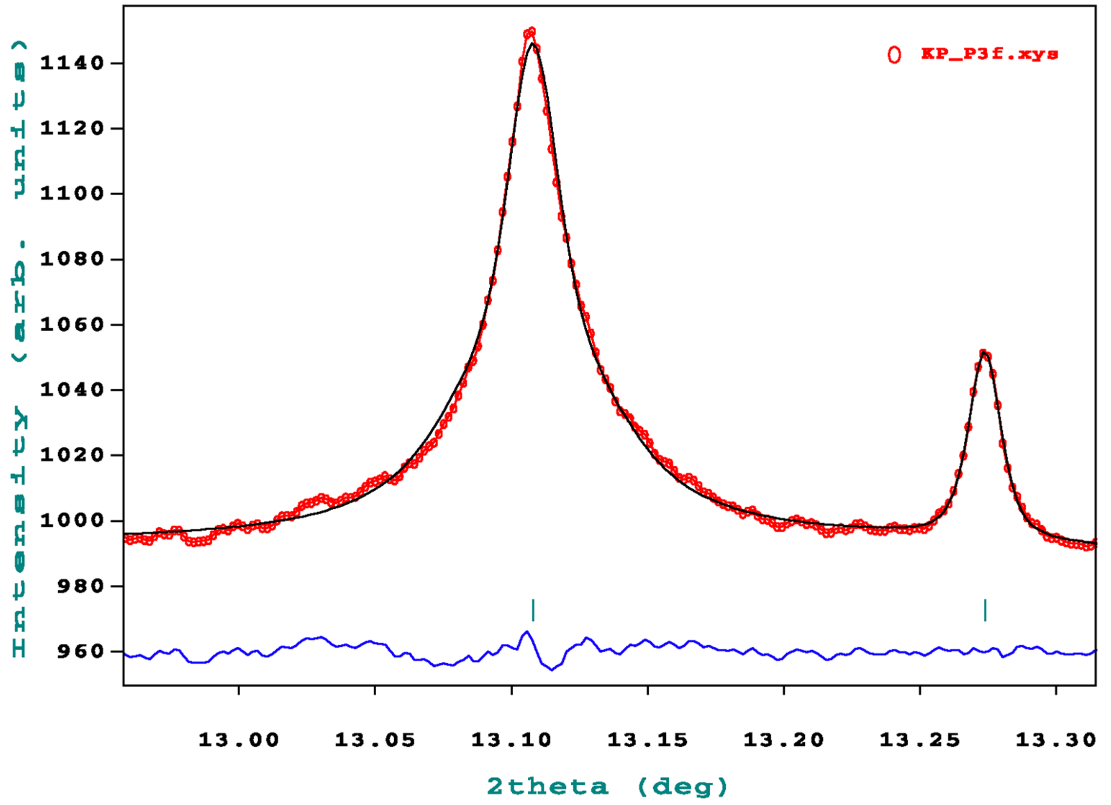

Figure S6.3. Comparison of reflections 311 and 410 from Le Bail (experiment #f21, top) and single peak fitting (bottom, $\chi^2 = 5.48$). Note the huge improvement with super-Lorentzian shape of 311 ($\eta = 1.29$). Red dots: observed intensities, black curve: calculated intensities, blue curve: difference; ticks for Bragg positions of kaliophilite are dark green.

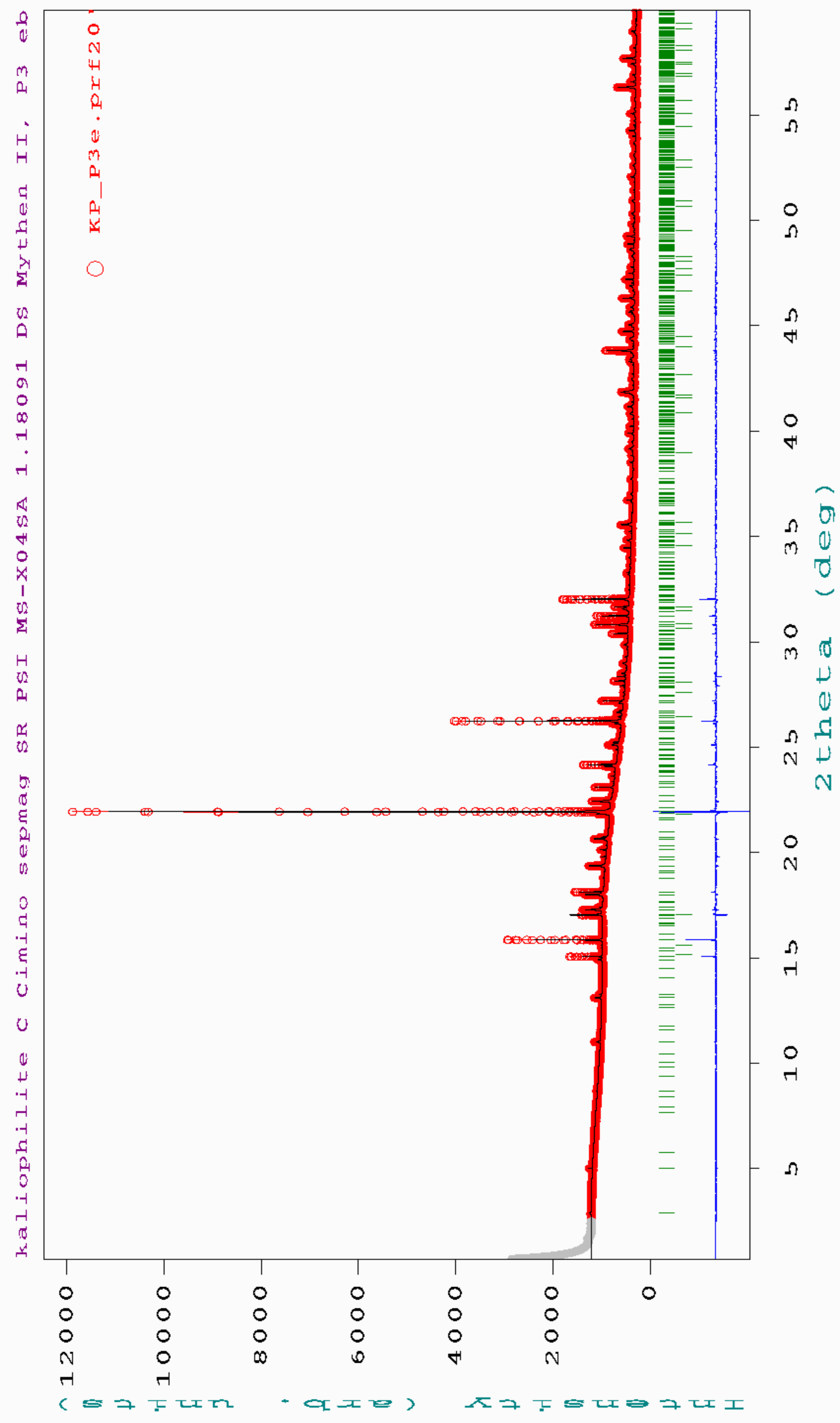

Figure S6.4. Rietveld fit #e20 for kaliophilite. Red dots: observed intensities, black curve: calculated intensities, blue curve: difference; ticks for Bragg positions are dark green for both kaliophilite and the kalsilite impurity.

Unit cell parameters of kaliophilite and their errors

For the Colle Cimino sample, the unit cell parameters obtained from Le Bail refinement #c18 are a = 27.0344(2), c = 8.56362(5) Å, V = 5420.28(9) Å^3 (experiment #f21 gave the same values but slightly lower errors). For the Monte Somma Vesuvius sample, the parameters were obtained from single crystal refinement and are different (a = 27.0597(16), c = 8.5587(6) Å).

Precisions are likely to be overestimated (Herbstein, 2000; David, 2004; Tian & Billinge, 2011), but there might also be some difference in composition and microstructure. The second phenomenon seems particularly interesting in view of the evident misfit of reflection 110 (Fig. S6.5). A test was therefore designed were reflection 110 was forced to fit observation (note that this reflection has a low intensity and little weight in the global refinement, but it occurs at low angle and is well separated from anything else). A good fit of the position was obtained for a = 27.051 Å which comes nearer to the Monte Somma value.

In this refinement, peak shape parameters stood almost unchanged at their original values, but the shift parameters changed and all unit cell parameters blew up. In particular, the sine shift parameter changed from 8.6 to 56.2 mdeg, which corresponds to sample displacements of about 0.1 and 0.8 mm along the X-ray beam. The first value comes near to the result for NAC standard refinement (realized at the same date) and is within the expected limits (Gozzo *et al.*, 2010), whereas the second value seems unreasonably high for the experimental setup at the MS-X04SA beamline.

We therefore postulate that the displacement of reflection 110 is due to details of the microstructure which are still to be resolved.

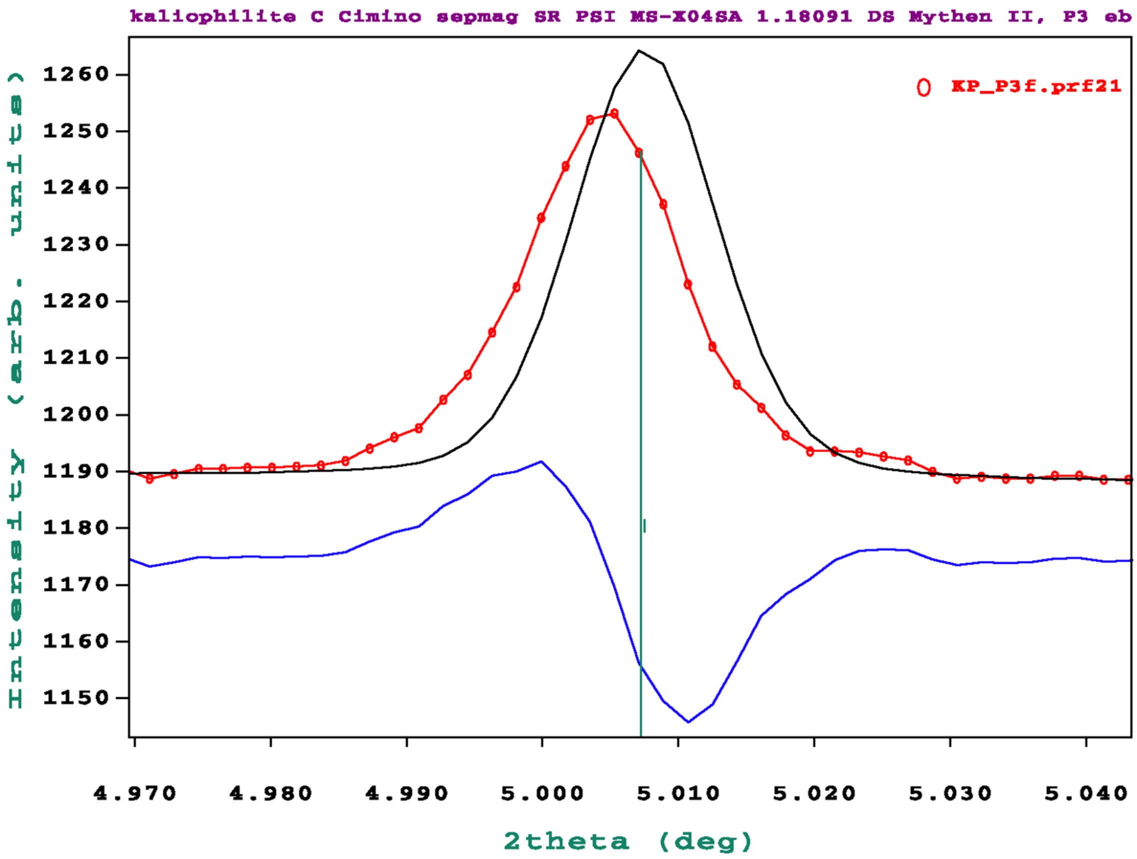

Figure S6.5. Reflection 110, obtained from Le Bail fitting (#f21). Note the shift of position. Red dots: observed intensities, black curve: calculated intensities, blue curve: difference; ticks for Bragg positions of kaliophilite are dark green.

References

David, W. I. F. (2004). *J. Appl. Cryst.* **37**, 621–628.

Gozzo, F., Cervellino, A., Leoni, M., Scardi, P., Bergamaschi, A. & Schmitt, B. (2010). *Z. Kristallogr.* **225**, 616–624.

Herbstein, F. H. (2000). *Acta Cryst.* B**56**, 547–557.

Larson, A. C. & Von Dreele, R. B. (2004). *General Structure Analysis System (GSAS)*, Los Alamos National Laboratory Report LAUR 86-748.

Rodríguez-Carvajal J. (2001). *IUCr Commission on Powder Diffraction Newsletter* **26**, 12–19.

Stephens, P. W. (1999). *J. Appl. Cryst.* **32**, 281–289.

Tian, P. & Billinge, S. J. L. (2011). *Z. Kristallogr.* **226**, 898–904.

S7. Pseudo-extinction rule

Mathematic derivation of systematic enhancement and weakening of reflections

The immediately striking features of both single-crystal electron and X-ray patterns are:

1) reflections $h - k = 3n$ are stronger than reflections $h - k \neq 3n$ for planes with l even;
2) reflections $h - k \neq 3n$ are stronger that reflections $h - k = 3n$ for planes with l odd;

These features may be intuitively associated with a non-crystallographic translation that becomes evident when describing the kaliophilite structure in terms of corollas containing seven six-membered rings.

The non-crystallographic translation **t** $=\left[\frac{1}{3}, -\frac{1}{3}, \frac{1}{2}\right]$ relates almost exactly the corollas centred on the ternaries in the unit cell interior (corollas A and B), but it is not valid between any one of these corollas and the corolla centred at the origin (corolla C). To prove the effect of this situation on diffraction intensities, we assume that the translation is valid for all atoms, i.e. that for each atom at coordinates x, y, z, a translated equal atom will occur at coordinates $x + \frac{1}{3}, y - \frac{1}{3}, z + \frac{1}{2}$.

For $2N$ atoms in the unit cell, the structure factor can then be written as

$$F_{hkl} = \sum_{j=1}^{N} f_j \exp[2\pi i(hx + ky + lz)] + \sum_{j=1}^{N} f_j \exp\left\{2\pi i \left[h\left(x + \frac{1}{3}\right) + k\left(y - \frac{1}{3}\right) + l\left(z + \frac{1}{2}\right)\right]\right\} =$$

$$= \sum f_j \exp[2\pi i(hx + ky + lz)] \left\{1 + exp\left[2\pi i\left(\frac{1}{3}h - \frac{1}{3}k + \frac{1}{2}l\right)\right]\right\}.$$

The second factor in brackets corresponds to $F_{hkl} = 0$ for $\left(\frac{1}{3}h - \frac{1}{3}k + \frac{1}{2}l\right) = \frac{2n+1}{2}$, and to a maximum of the structure factor for $\left(\frac{1}{3}h - \frac{1}{3}k + \frac{1}{2}l\right) = n.$

Let us separate two possible cases:

1) For $l = 2m$ the former condition (structure factor equal to zero) is never fulfilled for any values of h and k. It means that in general the reflections have intensities different from zero. However, the latter condition imposes that the reflections have maximum intensity for $h - k = 3n$.
2) For $l = 2m + 1$ the situation is reversed in the sense that $h - k = 3n$ corresponds to a null structure factor. The latter condition is never fulfilled, i.e. for $h - k \neq 3n$ we never obtain the maximum value, but intermediate ones.

These results explain the maximum intensities for hkl: $h - k = 3n$ and l even, and the almost zero intensities for hkl: $h - k = 3n$ and l odd, as observed in our experimental diffraction patterns.

We can also calculate in detail the relative values of all four groups of intensities when we simplify the problem to a trigonal unit cell containing only two atoms, at the positions $\frac{1}{3}$,-$\frac{1}{3}$, 0 and -$\frac{1}{3}$, $\frac{1}{3}$, $\frac{1}{2}$.

The structure factor can then be written as

$$F_{hkl} = f_j \left\{ \exp\left[i2\pi\left(h\frac{1}{3} - k\frac{1}{3} + l * 0\right)\right] + \exp\left[i2\pi\left(-h\frac{1}{3} + k\frac{1}{3} + l\frac{1}{2}\right)\right]\right\} \quad (1)$$

and

$$\frac{F_{hkl}}{f_j} = \exp\left[i2\pi\frac{1}{3}(h-k)\right] + \exp\left[-i2\pi\frac{1}{3}(h-k)\right]\exp(i\pi l). \quad (2)$$

For *l* even, equation (2) results in:

$$\frac{F_{hkl}}{f_j} = \exp\left[i2\pi\frac{1}{3}(h-k)\right] + \exp\left[-i2\pi\frac{1}{3}(h-k)\right]. \quad (3)$$

For *l* odd, equation (2) results in:

$$\frac{F_{hkl}}{f_j} = \exp\left[i2\pi\frac{1}{3}(h-k)\right] - \exp\left[-i2\pi\frac{1}{3}(h-k)\right]. \quad (4)$$

After substitution for parity rule $k = h - 3n$, equation (3) becomes:

$$\frac{F_{hkl}}{f_j} = \exp\left[i2\pi\frac{1}{3}(3n)\right] + \exp\left[-i2\pi\frac{1}{3}(3n)\right] = 2 \quad (5)$$

while equation (4) becomes:

$$\frac{F_{hkl}}{f_j} = \exp\left[i2\pi\frac{1}{3}(3n)\right] - \exp\left[-i2\pi\frac{1}{3}(3n)\right] = 0. \quad (6)$$

Conversely, for reflections that do not obey the parity rule, i.e. reflections for which $k = h - 3n \pm 1$, equation (3) becomes:

$$\frac{F_{hkl}}{f_j} = \exp\left[i2\pi\left(n \pm \frac{1}{3}\right)\right] + \exp\left[-i2\pi\left(n \pm \frac{1}{3}\right)\right] = -1 \quad (7)$$

while equation (4) becomes:

$$\frac{F_{hkl}}{f_j} = \exp\left[i2\pi\left(n \pm \frac{1}{3}\right)\right] - \exp\left[-i2\pi\left(n \pm \frac{1}{3}\right)\right] = \pm i\sqrt{3}. \quad (8)$$

Passing from structure factors to intensities, equations (5), (6), (7) and (8) show that, in layers with *l* even, the normalized intensities take the value 4 for reflections complying with the parity rule $h - k = 3n$, while intensities take value 1 for reflections not complying with such parity rule. Conversely, for layers with *l* odd, the normalized intensities take the value 0 for reflections complying with the parity rule $h - k = 3n$, while intensities take value 3 for reflections not complying with such parity rule. This is perfectly in agreement with the observed pseudo-extinctions or, more precisely, with the nearly fulfilled non-crystallographic extinctions of kaliophilite.
